# The serine synthesis pathway drives osteoclast differentiation through epigenetic regulation of NFATc1 expression

**DOI:** 10.1038/s42255-023-00948-y

**Published:** 2024-01-10

**Authors:** Steve Stegen, Karen Moermans, Ingrid Stockmans, Bernard Thienpont, Geert Carmeliet

**Affiliations:** 1 Laboratory of Clinical and Experimental Endocrinology, Department of Chronic Diseases and Metabolism, KU Leuven, Leuven, Belgium; 2 Laboratory of Functional Epigenetics, Department of Human Genetics, KU Leuven, Leuven, Belgium

**Keywords:** Osteoporosis, Molecular medicine, Mechanisms of disease, Metabolism

## Abstract

Bone-resorbing osteoclasts are vital for postnatal bone health, as increased differentiation or activity results in skeletal pathologies such as osteoporosis. The metabolism of mature osteoclasts differs from their progenitor cells, but whether the observed metabolic changes are secondary to the altered cell state or actively drive the process of cell differentiation is unknown. Here, we show that transient activation of the serine synthesis pathway (SSP) is essential for osteoclastogenesis, as deletion of the rate-limiting enzyme phosphoglycerate dehydrogenase in osteoclast progenitors impairs their differentiation and results in increased bone mass. In addition, pharmacological phosphoglycerate dehydrogenase inhibition abrogated bone loss in a mouse model of postmenopausal osteoporosis by blocking bone resorption. Mechanistically, SSP-derived α-ketoglutarate is necessary for histone demethylases that remove repressive histone methylation marks at the nuclear factor of activated T cells, cytoplasmic 1 (*Nfatc1*) gene locus, thereby inducing NFATc1 expression and consequent osteoclast maturation. Taken together, this study reveals a metabolic–epigenetic coupling mechanism that directs osteoclast differentiation and suggests that the SSP can be therapeutically targeted to prevent osteoporotic bone loss.

## Abstract (web summary)

Stegen et al. show that serine metabolism is transiently upregulated during osteoclastogenesis, and it drives osteoclast differentiation via epigenetic regulation of NFATc1 expression.

## Main

The importance of the skeleton is evident as structural and functional defects cause severe morbidities including impaired movement, disturbed mineral, hormonal and metabolic homeostasis, as well as deregulated haematopoiesis^1^. Maintaining bone homeostasis depends on a tightly regulated interplay between bone-forming osteoblasts and bone-resorbing osteoclasts. Changes in activity and differentiation capacity of skeletal cells are associated with different types of skeletal pathology, ranging from conditions of excessive bone formation to bone loss^2^. Osteoporosis is the most prevalent metabolic bone disease and is characterized by low bone mass, enhanced skeletal fragility and increased fracture risk^2,3^. Current therapies are mainly designed to block the increase in bone resorption during established osteoporosis but have not completely fulfilled their promise and are associated with adverse events^3,4^. Gaining more insight into the molecular mechanisms that govern osteoclast formation and activity is thus necessary to develop novel anti-osteoporosis drugs.

Bone-resorbing osteoclasts differentiate from monocytic precursor cells in the presence of macrophage colony-stimulating factor (M-CSF) and receptor activator of nuclear factor kappa-B ligand (RANKL)^5,6^. RANKL activates a complex signalling cascade that culminates in the induction of NFATc1, a critical pro-osteoclastogenic transcription factor^6,7^. Proper functioning of skeletal cells, including osteoclasts, depends not only on the activation of lineage-specific transcription factors, but also on a tailored metabolism^8–11^. Interestingly, gene expression and metabolism reciprocally regulate each other, as altered expression of metabolic enzymes in response to external stimuli leads to a specific metabolic profile that is needed to support cellular anabolism and function, whereas metabolic intermediates like acetyl-CoA, *S*-adenosyl methionine or α-ketoglutarate (αKG) are critical for DNA or histone-modifying enzymes to dynamically control the transcriptional programmes that control cell fate and differentiation^8,12–14^. Whether osteoclast precursors rely on specific metabolic pathways to initiate their typical gene expression profile during differentiation is however poorly studied.

In this study, we report that transient activation of de novo serine synthesis during early osteoclastogenesis is necessary to generate αKG, which is required to induce NFATc1 expression through epigenetic mechanisms and initiate osteoclast differentiation. Pharmacological inhibition of this metabolic pathway completely prevents oestrogen deficiency-induced bone loss by selectively blocking bone resorption, indicating that targeting osteoclast metabolism may be an appealing strategy to treat osteoporosis.

## Results

### Transient SSP activation during early osteoclastogenesis

**Fig. 1:**
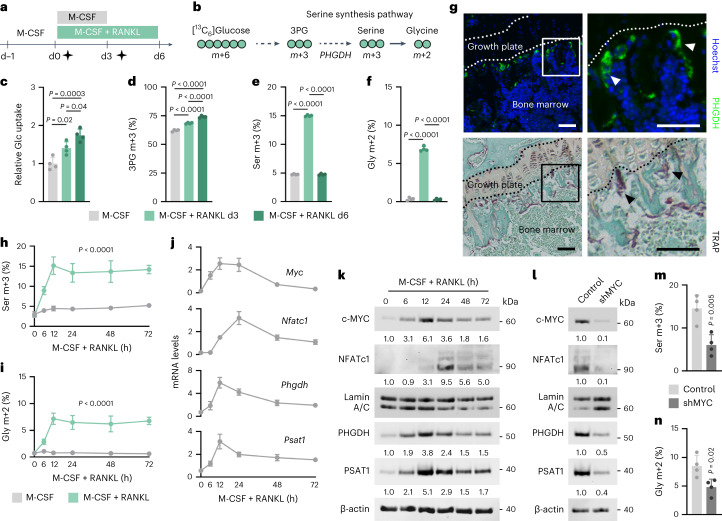
Transient activation of the SSP during early osteoclastogenesis. **a**, Schematic overview of the experimental setup for metabolomics analysis during osteoclastogenesis. [^13^C_6_]Glucose tracer was added (black stars) from day 0 (d0) to d3 (that is, early differentiation), or from d3 to d6 (that is, late differentiation). M-CSF-treated cells were used as baseline control. **b**, Schematic of carbon atom (circles) transitions of [^13^C_6_]glucose used to detect label incorporation in depicted metabolites. 3PG, 3-phosphoglycerate. **c**, Relative glucose (Glc) uptake at different timepoints during osteoclast differentiation (*n* = 4; one-way analysis of variance (ANOVA)). **d**–**f**, 3PG (**d**), serine (Ser; **e**) and glycine (Gly; **f**) labelling from [^13^C_6_]glucose at different timepoints during osteoclast differentiation (*n* = 4; one-way ANOVA). **g**, PHGDH immunostaining (upper) and TRAP staining (lower) of the long bone metaphysis of 8-week-old mice. Staining was performed on consecutive sections, and representative images from four mice are shown. Boxed areas are magnified, and arrowheads indicate osteoclasts. Scale bars, 50 µm. **h**,**i**, Ser (**h**) and Gly (**i**) labelling from [^13^C_6_]glucose at different timepoints during early osteoclast differentiation (*n* = 4; one-way ANOVA). **j**, *Myc*, *Nfatc1*, *Phgdh* and *Psat1* mRNA levels at different timepoints during early osteoclast differentiation (*n* = 4). Expression levels were calculated relative to *Hprt* expression. **k**, c-MYC, NFATc1, PHGDH and PSAT1 protein levels at different timepoints during early osteoclast differentiation. Lamin A/C and β-actin were used as loading controls for nuclear and cytoplasmic proteins, respectively. Representative images from three independent experiments are shown. **l**, c-MYC, NFATc1, PHGDH and PSAT1 protein levels in control (empty vector) or c-MYC-silenced (shRNA against c-MYC) osteoclasts, 3 d after transduction. Lamin A/C and β-actin were used as loading controls for nuclear and cytoplasmic proteins, respectively. Representative images from three independent experiments are shown. **m**,**n**, Ser (**m**) and Gly (**n**) labelling from [^13^C_6_]glucose in control or c-MYC-silenced osteoclasts (*n* = 4; unpaired, two-tailed Student’s *t*-test). Data are means ± s.d. Relevant MDVs are depicted in **d**–**f**, **h**, **i**, **m** and **n**. Source data

**Extended Data Fig. 1:**
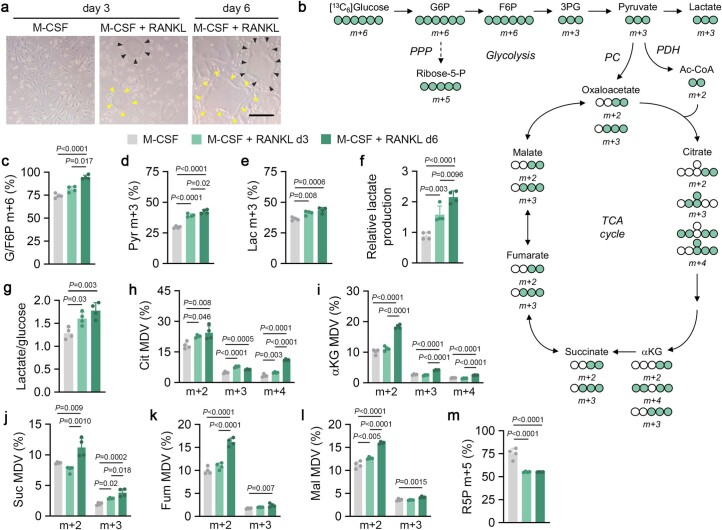
Analysis of glucose metabolism during osteoclast differentiation. **a** Representative pictures of osteoclast differentiation at indicated timepoints. Black and yellow arrowheads indicate osteoclasts. Scale bar is 50 µm. Representative pictures of 4 biological replicates are shown. **b** Schematic of carbon atom (circles) transitions of [^13^C_6_]glucose used to detect label incorporation into indicated metabolites. G6P is glucose-6-phosphate, F6P is fructose-6-phosphate, 3PG is 3-phosphoglycerate, Ac-CoA is acetyl-CoA, aKG is α-ketoglutarate. **c-e** G6P/F6P (**c**), pyruvate (Pyr; **d**) lactate (Lac; **e**) labeling from [^13^C_6_]glucose at different timepoints during osteoclast differentiation (n = 4; one-way ANOVA). **f**, **g** Relative lactate production (**f**) and ratio of lactate production over glucose consumption (**g**) (n = 4; one-way ANOVA). **h-m** Citrate (Cit; **h**), aKG (**i**), succinate (Suc; **j**), fumarate (Fum; **k**), malate (Mal; **l**) and ribose-5-phosphate (R5P; **m**) labeling from [^13^C_6_]glucose (n = 4; one-way ANOVA). Data are means ± s.d. Relevant mass distribution vectors (MDVs) are shown in (**c-e**) and (**h-m**). Source data

To study whether osteoclast progenitors have a specific metabolic profile that drives their differentiation, we performed isotopic labelling during the differentiation of bone marrow mononuclear cells (BMMCs) into mature osteoclasts. In this model, BMMC differentiation is induced using M-CSF and RANKL^15^. BMMCs were cultured in the presence of [^13^C_6_]glucose, a critical nutrient during osteoclastogenesis^16^, either during the first 3 d of RANKL treatment (that is, early differentiation) or from day 3 to day 6 (that is, late differentiation) and compared to M-CSF-treated BMMCs at day 3 (Fig. 1a and Extended Data Fig. 1a). Consistent with previous reports^16–19^, we noticed a gradual increase in glucose consumption during osteoclast differentiation, which was associated with enhanced glycolysis, as evidenced by increased ^13^C labelling of glycolytic intermediates and lactate production (Fig. 1b–d and Extended Data Fig. 1b–g). Glucose-dependent fuelling of the tricarboxylic acid (TCA) cycle was mainly via pyruvate dehydrogenase (m + 2, m + 4 mass distribution vectors (MDVs)) and less via pyruvate carboxylase (m + 3 MDV) and it increased predominantly during late differentiation (Extended Data Fig. 1h–l), likely to support the enhanced oxidative phosphorylation that is observed in mature osteoclasts^18–20^.

In addition to the changes in glycolysis and glucose-dependent TCA cycle anaplerosis, we also noticed alterations in other glucose-derived metabolic pathways. [^13^C]Glucose labelling of ribose-5-phosphate, the pentose phosphate pathway intermediate that supports nucleotide synthesis, was reduced after RANKL treatment (Extended Data Fig. 1m), consistent with findings that proliferation is decreased in differentiating osteoclasts^21^. Interestingly, we found that the SSP, which generates the amino acids serine and glycine de novo from the glycolytic intermediate 3-phosophoglycerate^22^, was transiently but manifestly upregulated during osteoclast differentiation, with a peak at day 3 and returning to baseline by day 6 (Fig. 1b,e,f). In line with this, we noticed that many, but not all, bone-lining osteoclasts expressed the rate-limiting SSP enzyme phosphoglycerate dehydrogenase (PHGDH; Fig. 1g). Based on these metabolic analyses, we further investigated the functional importance of de novo serine synthesis, because its role in osteoclasts is completely unknown.

**Extended Data Fig. 2:**
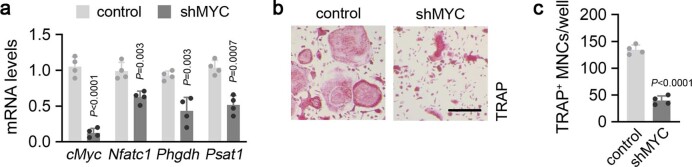
Genetic silencing of c-MYC impairs osteoclastogenesis and serine synthesis pathway-related gene expression. **a** *cMyc*, *Nfatc1*, *Phgdh*, *Psat1* mRNA levels in osteoclasts after transduction with a lentiviral vector carrying a shRNA against c-MYC (shMYC) (n = 4; unpaired, two-tailed Student’s *t*-test). An empty vector was used as control. **b**, **c** TRAP staining (**b**) and quantification of control and c-MYC-silenced TRAP^+^ multinuclear cells (MNCs; **c**) (n = 4 biologically independent samples; unpaired, two-tailed Student’s *t*-test). Scale bar is 50 µm. Data are means ± s.d. Source data

By investigating the metabolic dynamics of SSP activation in more detail, we observed that already 6 h after RANKL treatment, glucose-derived carbon contribution to serine and glycine was increased and peaked at the 12-h timepoint (Fig. 1h,i). These metabolic changes were confirmed by a similar gene and protein expression pattern of the SSP-related enzymes PHGDH and phosphoserine aminotransferase 1 (PSAT1; Fig. 1j,k). Intriguingly, activation of the SSP occurred before the induction of NFATc1, suggesting that this metabolic pathway contributes to proper osteoclast lineage allocation. Therefore, we explored the molecular pathways that couple RANKL signalling to de novo serine synthesis. In tumour cells, the transcription factor c-MYC activates the SSP by directly inducing the expression of *Phgdh* and *Psat1* (ref. ^23^). Because c-MYC regulates osteoclastogenesis by inducing *Nfatc1* expression^24–26^ and rewiring osteoclast metabolism^27^, we questioned whether c-MYC and the SSP are linked during osteoclastogenesis. We observed that c-MYC levels gradually increased in the first 12 h after RANKL treatment, thereby parallelling the dynamics in PHGDH and PSAT1 expression (Fig. 1j,k). Importantly, shRNA-mediated silencing of c-MYC in osteoclast precursors decreased PHGDH and PSAT1 levels and [^13^C]glucose incorporation in serine and glycine, next to reducing NFATc1 expression and osteoclast differentiation (Fig. 1l–n and Extended Data Fig. 2a–c). Together, these data indicate that during early osteoclast differentiation RANKL signalling transiently stimulates the SSP via c-MYC-mediated transcription of the SSP-related enzymes PHGDH and PSAT1.

### PHGDH loss in osteoclasts impairs bone resorption and increases bone mass

**Fig. 2:**
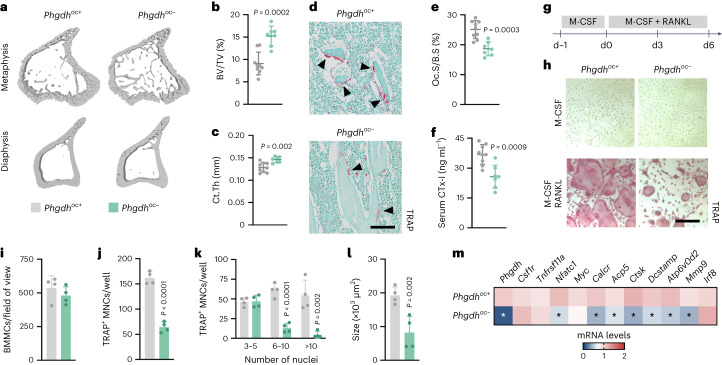
PHGDH in osteoclasts controls bone resorption and postnatal bone mass. **a**–**c**, Three-dimensional (3D) micro-CT models (**a**) of the tibial metaphysis (top) and diaphysis (bottom) with quantification of trabecular bone volume (BV/TV; **b**) and cortical thickness (Ct.Th; **c**) in wild-type (*Phgdh*^*oc+*^) and osteoclast-specific PHGDH knockout (*Phgdh*^*oc*−^) mice (*n* = 9 *Phgdh*^*oc+*^ and 7 *Phgdh*^*oc*−^ mice; unpaired, two-tailed Student’s *t*-test). **d**,**e**, TRAP staining of the tibial metaphysis (**d**) with quantification (**e**) of osteoclast surface per bone surface (Oc.S/B.S) in *Phgdh*^*oc+*^ and *Phgdh*^*oc*−^ mice (*n* = 9 *Phgdh*^*oc+*^ and 7 *Phgdh*^*oc*−^ mice; unpaired, two-tailed Student’s *t*-test). Scale bar, 100 µm. **f**, Serum CTx-I levels in *Phgdh*^*oc+*^ and *Phgdh*^*oc*−^ mice (*n* = 9 *Phgdh*^*oc+*^ and 7 *Phgdh*^*oc*−^ mice; unpaired, two-tailed Student’s *t*-test). **g**, Schematic overview of the experimental setup used to study osteoclast differentiation. **h**, TRAP staining of osteoclast progenitors (upper) and osteoclasts (lower) from *Phgdh*^*oc+*^ and *Phgdh*^*oc*−^ mice treated with M-CSF alone or M-CSF and RANKL, respectively (*n* = 4). Scale bar, 50 µm. **i**–**l**, Number of BMMCs (**i**), total number of TRAP-positive (TRAP^+^) MNCs (**j**), number of TRAP^+^ MNCs with indication of number of nuclei per cell (**k**) and average size of TRAP^+^ MNCs (**l**) from *Phgdh*^*oc+*^ and *Phgdh*^*oc*−^ mice after M-CSF and RANKL treatment (*n* = 4; all unpaired, two-tailed Student’s *t*-test). **m**, *Phgdh* (*P* < 0.0001), *Csf1r*, *Tnfrsf11a* (encoding RANK), *Nfatc1* (*P* = 0.002), *Myc*, *Calcr* (*P* = 0.0002), *Acp5* (*P* = 0.008), *Ctsk* (*P* = 0.0002), *Dcstamp* (*P* = 0.0016), *Atp6v0d2* (*P* = 0.007), *Mmp9* (*P* = 0.010) and *Irf8* mRNA levels in cultured osteoclasts from *Phgdh*^*oc+*^ and *Phgdh*^*oc*−^ mice after M-CSF and RANKL treatment (*n* = 4; unpaired, two-tailed Student’s *t*-test with *P < 0.05 versus *Phgdh*^*oc+*^). Data are means ± s.d. Source data

**Extended Data Fig. 3:**
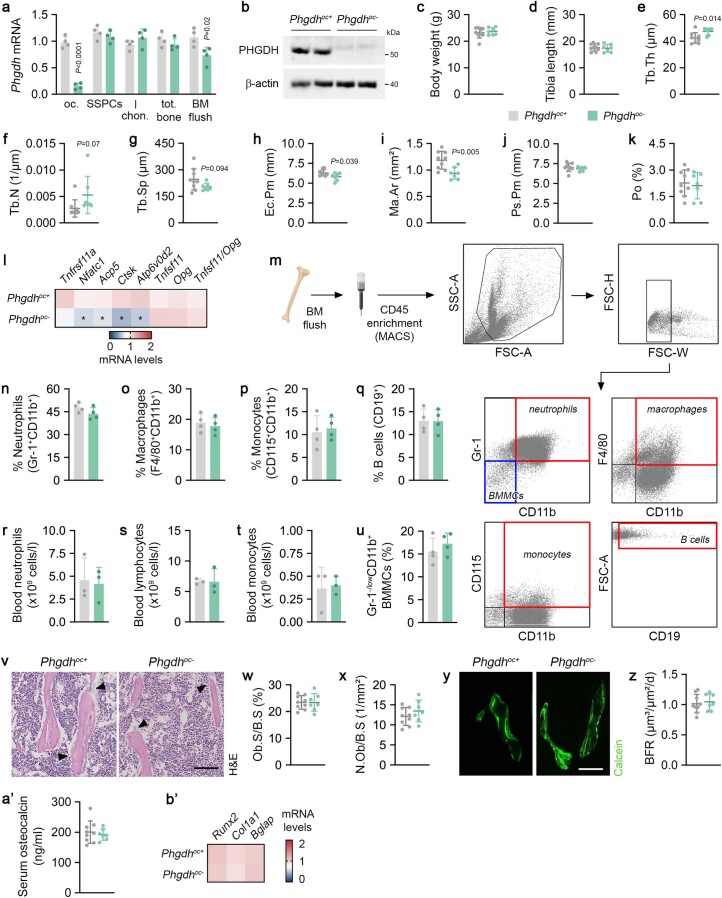
Phenotype of osteoclast-specific PHGDH knockout mice. **a** *Phgdh* mRNA levels in osteoclasts (oc.), skeletal stem/progenitor cells (SSPCs), chondrocytes (chon.), total bone (tot. bone) and bone marrow cells obtained after flushing (BM flush) from wild-type (*Phgdh*^*oc+*^) and osteoclast-specific PHGDH knockout (*Phgdh*^*oc-*^) mice (n = 4; unpaired, two-tailed Student’s *t*-test). **b** Immunoblot of PHGDH and β-actin levels in cultured osteoclasts isolated from *Phgdh*^*oc+*^ and *Phgdh*^*oc-*^ mice (n = 4). Two biological replicates are shown. **c**, **d** Body weight (**c**) and tibia length (**d**) of *Phgdh*^*oc+*^ and *Phgdh*^*oc-*^ male mice (n = 9 *Phgdh*^*oc+*^ and 7 *Phgdh*^*oc-*^ mice). **e-k** MicroCT-based analysis of trabecular thickness (Tb.Th; **e**), trabecular number (Tb.N; **f**), trabecular separation (Tb.Sp; **g**), endocortical perimeter (Ec.Pm; **h**), medullary area (Ma.Ar; **i**), periosteal perimeter (Ps.Pm; **j**) and cortical porosity (Po; **k**) in tibiae from *Phgdh*^*oc+*^ and *Phgdh*^*oc-*^ mice (n = 9 *Phgdh*^*oc+*^ and 7 *Phgdh*^*oc-*^ mice; unpaired, two-tailed Student’s *t*-test). **l** *Tnfrsf11a* (encoding for RANK), *Nfatc1* (*P* = 0.04), *Acp5* (*P* = 0.046), *Ctsk* (*P* = 0.007), *Atp6v0d2* (*P* = 0.0008), *Tnfsf11* (encoding for RANKL), *Opg* and *Tnfsf11*/*Opg* mRNA levels in total bone from *Phgdh*^*oc+*^ and *Phgdh*^*oc-*^ mice (n = 4; unpaired, two-tailed Student’s *t*-test with *p < 0.05 vs *Phgdh*^*oc+*^). **m** Schematic overview of the experimental setup for isolation of BM cells that are enriched for CD45 expression, with gating strategy for flow cytometry analysis of BM-derived myeloid subpopulations. BMMCs is bone marrow mononuclear cells. Created with BioRender.com. **n-q** Quantification of the number of neutrophils (**n**), macrophages (**o**), monocytes (**p**) and B cells (**q**) as percent of the CD45-positive BM population (n = 4). **r-t** Quantification of circulating neutrophils (**r**), lymphocytes (**s**) and monocytes (**t**) in *Phgdh*^*oc+*^ and *Phgdh*^*oc-*^ mice (n = 3). **u** Quantification of the number of Gr-1^-/low^CD11b^+^ BMMCs as percentage of the CD45-positive BM population of *Phgdh*^*oc+*^ and *Phgdh*^*oc-*^ mice (n = 4). **v-x** H&E staining of the tibial metaphysis (**v**) with quantification of osteoblast surface per bone surface (Ob.S/B.S; **w**) and the number of osteoblasts per bone surface (N.Ob/B.S; **x**) in *Phgdh*^*oc+*^ and *Phgdh*^*oc-*^ mice (n = 9 *Phgdh*^*oc+*^ and 7 *Phgdh*^*oc-*^ mice). Scale bar is 100 µm. **y-z** Calcein staining of the tibial metaphysis (**y**) with quantification of the bone formation rate (BFR; **z**) in *Phgdh*^*oc+*^ and *Phgdh*^*oc-*^ mice (n = 9 *Phgdh*^*oc+*^ and 7 *Phgdh*^*oc-*^ mice). Scale bar is 100 µm. **a**′**-b**′ Serum osteocalcin levels (**a**′; n = 9 *Phgdh*^*oc+*^ and 7 *Phgdh*^*oc-*^ mice) and *Runx2*, *Col1a1* and *Bglap* mRNA levels (**b**′) in flushed bone from *Phgdh*^*oc+*^ and *Phgdh*^*oc-*^ mice (n = 4). Data are means ± s.d. Source data

To determine whether the activation of the SSP during early osteoclastogenesis has a physiological role during bone homeostasis, we deleted the rate-limiting enzyme PHGDH by crossing *Phgdh*^*lox/lox*^ mice with *lysozyme 2*-Cre transgenic mice (*Phgdh*^*oc*−^), resulting in specific and efficient deletion of PHGDH at the mRNA and protein level (Extended Data Fig. 3a,b). *Phgdh*^*oc*−^ mice were viable and undistinguishable from control littermates at birth and showed normal growth, as body mass and tibia length were comparable to control mice (Extended Data Fig. 3c,d and Supplementary Table 1). However, bone mass was substantially increased in male and female mutant mice at 8 weeks of age, as evidenced by ex vivo micro-computed tomography (micro-CT; Fig. 2a–c and Supplementary Table 1). Trabecular bone volume was almost twofold increased, which was predominantly caused by an increase in trabecular thickness, resulting in decreased trabecular separation (Fig. 2b and Extended Data Fig. 3e–g). Moreover, cortical thickness was increased by ~15%, which was related to a decreased endocortical perimeter together with a reduction in medullary area (Fig. 2c and Extended Data Fig. 3h,i). Periosteal perimeter and cortical porosity were not affected in mutant mice (Extended Data Fig. 3j,k). Taken together, our data indicate that osteoclastic PHGDH regulates postnatal bone homeostasis.

**Extended Data Fig. 4:**
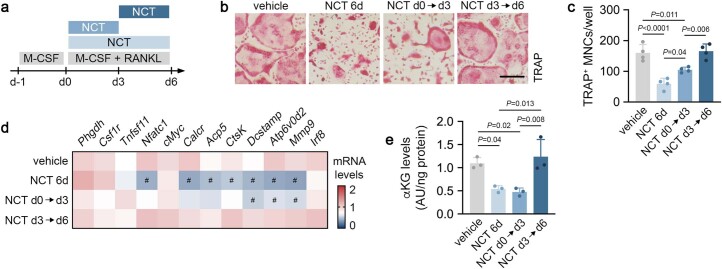
Pharmacological PHGDH inhibition during early differentiation decreases osteoclastogenesis. **a** Schematic overview of experimental setup. Osteoclast precursors were treated with the PHGDH inhibitor NCT-503 (NCT) either during the first three days (d0 to d3), the last three days (d3 to d6) or during the entire differentiation. **b**, **c** TRAP staining (**b**) and quantification of TRAP^+^ MNCs (**c**) from wild-type mice treated either with vehicle or NCT-503 (n = 4; one-way ANOVA). Scale bar is 50 µm. **d** *Phgdh*, *Csf1r*, *Tnfrsf11a* (encoding for RANK), *Nfatc1* (*P* = 0.0018), *cMyc*, *Calcr* (*P* = 0.002), *Acp5* (*P* = 0.02), *Ctsk* (*P* = 0.005), *Dcstamp* (NCT 6d: *P* = 0.002; NCT d0 to d3: *P* = 0.04), *Atp6v0d2* (NCT 6d: *P* < 0.0001; NCT d0 to d3: *P* = 0.0008), *Mmp9* (NCT 6d: *P* = 0.0013; NCT d0 to d3: *P* = 0.010) and *Irf8* mRNA levels in osteoclasts treated with either vehicle or NCT-503 (n = 4; one-way ANOVA with ^#^p < 0.05 vs vehicle). **e** αKG levels in osteoclasts treated with either vehicle or NCT-503 (n = 3; one-way ANOVA). Data are means ± s.d. Source data

To verify our hypothesis that bone resorption was impaired in *Phgdh*^*oc*−^ mice, we analysed osteoclast parameters. Histomorphometry of tartrate-resistant acid phosphatase (TRAP)-stained sections revealed a decrease in osteoclast surface, and the reduced osteoclast-related gene expression in total bone and serum CTx-I levels further confirmed reduced osteoclast formation and activity in mutant bones (Fig. 2d–f and Extended Data Fig. 3l). Other myeloid cell types were not affected in *Phgdh*^*oc*−^ mice (Extended Data Fig. 3m–t). The importance of PHGDH for osteoclastogenesis was also recapitulated during in vitro differentiation. While M-CSF-treated mutant BMMCs did not display an apparent functional phenotype (Extended Data Fig. 3u), we observed a manifest reduction in the number of large, TRAP-positive multinucleated cells after stimulation with RANKL, which was associated with a decrease in osteoclast marker gene expression (Fig. 2g–m). Consistent with the PHGDH expression profile (Fig. 1j,k), the activation of the SSP is only transiently needed during early differentiation, as pharmacological PHGDH inhibition during the first 3 d reduced osteoclast differentiation, whereas inhibition between day 3 and day 6 had no effect (Extended Data Fig. 4a–d).

In contrast to the profound reduction in bone resorption parameters, bone formation was not affected in *Phgdh*^*oc*−^ mice, as we observed no quantitative changes in osteoblast number or activity in vivo, serum osteocalcin levels or osteoblast marker gene expression (Extended Data Fig. 3v-b′). Taken together, the SSP, through PHGDH, is required for osteoclastogenesis during bone homeostasis.

### PSAT1-derived αKG is crucial for osteoclast differentiation

**Fig. 3:**
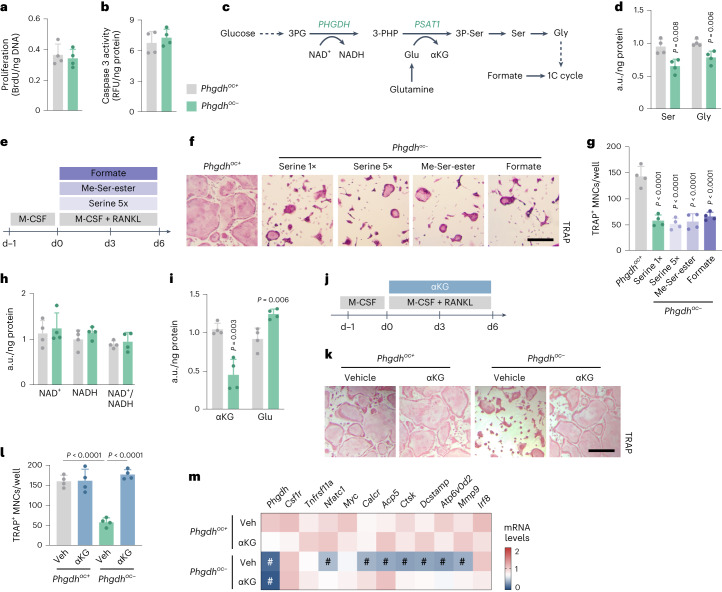
SSP-derived αKG supports osteoclast differentiation. **a**,**b**, Proliferation, measured by 5′-bromo-2′-deoxyuridine (BrdU) incorporation (**a**), and survival, measured via caspase 3 activity (**b**), of cultured osteoclasts from *Phgdh*^*oc+*^ and *Phgdh*^*oc*−^ mice (*n* = 4). **c**, Schematic overview of de novo serine synthesis and its by-products. 1C cycle, one-carbon cycle; 3-PHP, 3-phosphohydroxypyruvate; 3P-Ser, 3-phosphoserine. **d**, Intracellular Ser and Gly levels in osteoclasts from *Phgdh*^*oc+*^ and *Phgdh*^*oc*−^ mice (*n* = 4; unpaired, two-tailed Student’s *t*-test). **e**, Schematic overview of the experimental setup. Osteoclasts were treated during the entire experiment. Me-Ser-ester, methyl-serine-ester; serine 5×, 5 times the Ser concentration that is normally present in the culture medium (that is, final concentration of 1.2 mM). **f**,**g**, TRAP staining (**f**) and quantification of TRAP^+^ MNCs (**g**) from *Phgdh*^*oc+*^ and *Phgdh*^*oc*−^ mice treated, in addition to M-CSF and RANKL, either with vehicle, Me-Ser-ester or formate, or cultured in 5× serine levels (*n* = 4; one-way ANOVA). **h**, Intracellular NAD(H) species in osteoclasts from *Phgdh*^*oc+*^ and *Phgdh*^*oc*−^ mice (*n* = 4). **i**, Intracellular αKG and glutamate (Glu) levels in osteoclasts from *Phgdh*^*oc+*^ and *Phgdh*^*oc*−^ mice (*n* = 4; unpaired, two-tailed Student’s *t*-test). **j**, Schematic overview of experimental setup. Osteoclasts were treated with cell-permeable αKG during differentiation. **k**,**l**, TRAP staining (**k**) and quantification of TRAP^+^ MNCs (**l**) from *Phgdh*^*oc+*^ and *Phgdh*^*oc*−^ mice treated either with vehicle or αKG (*n* = 4; two-way ANOVA). **m**, *Phgdh* (*Phgdh*^*oc–*^veh: *P* < 0.0001; *Phgdh*^*oc–*^αKG: *P* < 0.0001), *Csf1r*, *Tnfrsf11a* (encoding RANK), *Nfatc1* (*P* = 0.0004), *Myc*, *Calcr* (*P* = 0.04), *Acp5* (*P* = 0.008), *Ctsk* (*P* = 0.0006), *Dcstamp* (*P* = 0.03), *Atp6v0d2* (*P* = 0.004), *Mmp9* (*P* = 0.012) and *Irf8* mRNA levels in osteoclasts from *Phgdh*^*oc+*^ and *Phgdh*^*oc*−^ mice treated with either vehicle or αKG (*n* = 4; two-way ANOVA with ^#^*P* < 0.05 versus *Phgdh*^*oc+*^- veh). Data are means ± s.d. Scale bars, 50 µm (**f** and **k**). a.u., arbitrary units; RFU, relative fluorescence unit. Source data

**Extended Data Fig. 5:**
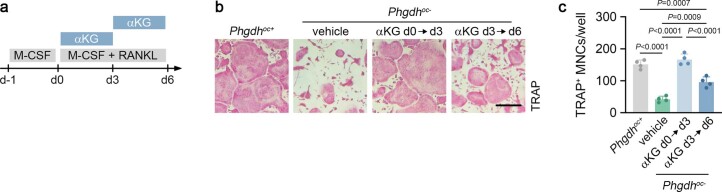
Supplementation of αKG rescues the defect in osteoclastogenesis in PHGDH-deficient cells. **a** Schematic overview of experimental setup. Osteoclast precursors were treated with cell-permeable αKG either during the first three days (d0 to d3) or during the last three days (d3 to d6) of differentiation. **b**, **c** TRAP staining (**b**) and quantification of TRAP^+^ MNCs (**c**) from *Phgdh*^*oc+*^ and *Phgdh*^*oc-*^ mice treated with vehicle or αKG (n = 4; one-way ANOVA). Scale bar is 50 µm. Data are means ± s.d. Source data

Osteoclast formation was reduced upon PHGDH deletion and was primarily caused by defective differentiation with decreased osteoclastogenic gene expression, whereas proliferation and cell viability were not affected (Fig. 3a,b). To further understand how the SSP metabolically regulates osteoclast differentiation, we focused on the SSP-derived amino acids serine and glycine, and on SSP-derived metabolic by-products like NADH generated by PHGDH or αKG produced by PSAT1, an enzyme downstream of PHGDH (Fig. 3c). *Phgdh* deletion reduced intracellular levels of serine and glycine at day 3 of osteoclastogenesis (Fig. 3d) but administering additional serine or a cell-permeable methyl-serine-ester or the downstream SSP metabolite formate failed to rescue osteoclast differentiation (Fig. 3e–g), indicating that SSP-derived amino acids and one-carbon units are not a limiting factor. In addition, intracellular NAD^+^ and NADH levels, as well as the NAD^+^/NADH ratio, were not altered in mutant osteoclasts, excluding NADH metabolism as an explanation (Fig. 3h). By contrast, αKG levels were strongly downregulated, whereas glutamate levels were increased (Fig. 3i), which may be explained by reduced PSAT1 activity (Fig. 3c). A similar effect on αKG levels was observed following pharmacological PHGDH inhibition using NCT-503 (Extended Data Fig. 4e). As further evidence of the importance of αKG, exogenous cell-permeable αKG was sufficient to induce osteoclast differentiation in PHGDH-deficient cells, with complete normalization of osteoclast-related gene expression (Fig. 3j–m). The functional rescue by αKG fully coincided with activation of the SSP during early osteoclastogenesis, as αKG supplementation during the first 3 d completely rescued the defect in differentiation, whereas αKG treatment from day 3 to day 6 only had a mild effect (Extended Data Fig. 5a–c). Thus, the SSP is essential for osteoclastogenesis as PSAT1 generates αKG that is necessary for proper differentiation.

**Fig. 4:**
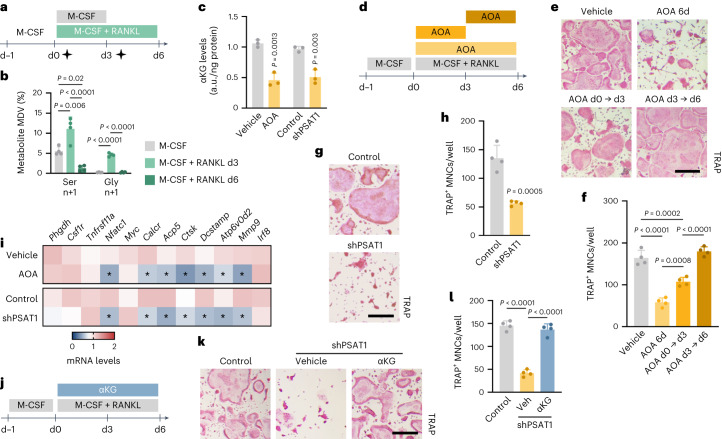
The SSP enzyme PSAT1 generates αKG during early osteoclastogenesis. **a**, Schematic overview of experimental setup for metabolomics analysis during osteoclastogenesis. [^13^C_5_/^15^N_2_]glutamine tracer was added (black stars) from d0 to d3 (that is, early differentiation), or from d3 to d6 (that is, late differentiation). **b**, Ser and Gly labelling from [^13^C_5_/^15^N_2_]glutamine at different timepoints during osteoclast differentiation (*n* = 4; one-way ANOVA). Relevant MDVs are shown; n denotes specific labelling from nitrogen. **c**, Intracellular αKG levels in vehicle and AOA-treated osteoclasts, or in control and PSAT1-silenced (shPSAT1) osteoclasts (*n* = 4; unpaired, two-tailed Student’s *t*-test). An empty vector was used as shRNA control. **d**, Schematic overview of experimental setup. Osteoclasts were treated with AOA during the first 3 d, the last 3 d or during the entire experiment. **e**,**f**, TRAP staining (**e**) and quantification of TRAP^+^ MNCs (**f**) from wild-type mice treated with either vehicle or AOA (*n* = 4; one-way ANOVA). **g**,**h**, TRAP staining (**g**) and quantification of TRAP^+^ MNCs (**h**) after shRNA-mediated silencing of PSAT1 (*n* = 4 biologically independent samples; unpaired, two-tailed Student’s *t*-test). An empty vector was used as control. **i**, *Phgdh*, *Csf1r*, *Tnfrsf11a* (encoding RANK), *Nfatc1* (AOA: *P* = 0.002; shPSAT1: *P* = 0.008), *Myc*, *Calcr* (AOA: *P* = 0.006; shPSAT1: *P* = 0.013), *Acp5* (AOA: *P* = 0.03; shPSAT1: *P* = 0.007), *Ctsk* (AOA: *P* < 0.0001; shPSAT1: *P* = 0.048), *Dcstamp* (AOA: *P* = 0.0095; shPSAT1: *P* = 0.013), *Atp6v0d2* (AOA: *P* = 0.04; shPSAT1: *P* = 0.011), *Mmp9* (AOA: *P* = 0.005; shPSAT1: *P* = 0.04) and *Irf8* mRNA levels in AOA-treated or PSAT1-silenced osteoclasts (*n* = 4; unpaired, two-tailed Student’s *t*-test with *P < 0.05 versus vehicle/control). **j**, Schematic overview of experimental setup. PSAT1-silenced osteoclasts were treated with cell-permeable αKG during the entire experiment. **k**,**l**, TRAP staining (**k**) and quantification of control and αKG-treated PSAT1-silenced TRAP^+^ MNCs (**l**) (*n* = 4 biologically independent samples; one-way ANOVA). Data are means ± s.d. Scale bars, 50 µm (**e**, **g** and **k**). Source data

**Extended Data Fig. 6:**
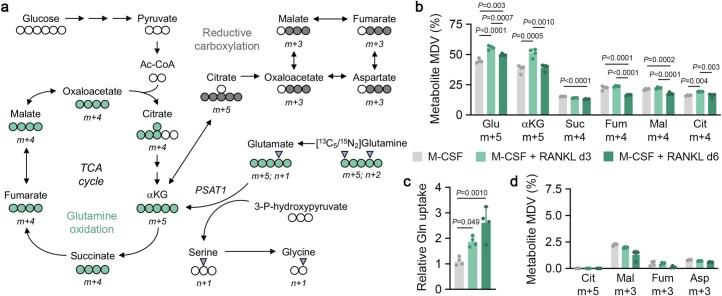
[^13^C_5_/^15^N_2_]Glutamine tracing analysis during osteoclast differentiation. **a** Schematic of carbon (circles) and nitrogen (triangles) atom transitions of [^13^C_5_/^15^N_2_]glutamine used to detect label incorporation into indicated metabolites. **b** [^13^C_5_/^15^N_2_]Glutamine labeling of glutamate (Glu), α-ketoglutarate (αKG), succinate (Suc), fumarate (Fum), malate (Mal) and citrate (Cit) via glutamine oxidation (n = 4; one-way ANOVA). **c** Relative glutamine (Gln) uptake (n = 4; one-way ANOVA). **d** [^13^C_5_/^15^N_2_]Glutamine labeling of indicated metabolites via reductive carboxylation (n = 4). Asp is aspartate. Data are means ± s.d. Relevant mass distribution vectors (MDVs) are shown in (**b**) and (**d**). Source data

**Extended Data Fig. 7:**
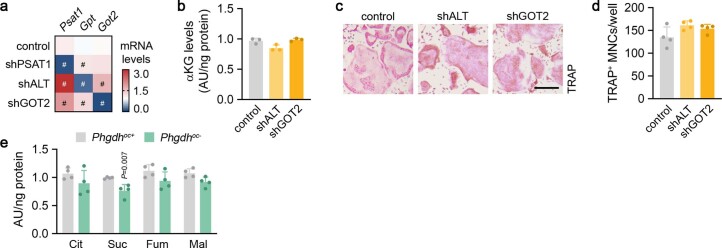
Impact of ALT or GOT2 silencing on osteoclastogenesis and effect of PHGDH deletion on TCA cycle intermediates. **a** *Psat1* (shPSAT1: *P* = 0.007; shALT: *P* < 0.0001; shGOT2: *P* = 0.007), *Gpt* (encoding for ALT; shPSAT1: *P* = 0.020; shALT: *P* < 0.0001; shGOT2: *P* = 0.0007) and *Got2* (shALT: *P* = 0.0094; shGOT2: *P* = 0.0004) mRNA levels in osteoclasts transduced with a lentivirus carrying an empty vector (control) or a shRNA against PSAT1, ALT or GOT2 (n = 4; one-way ANOVA with ^#^p < 0.05 vs control). **b** Intracellular αKG levels in osteoclasts after shRNA-mediated silencing of ALT or GOT2 (n = 3). **c, d** TRAP staining (**c**) and quantification of TRAP^+^ MNCs after shRNA-mediated silencing of ALT or GOT2 (**d**) (n = 4 biologically independent samples). Scale bar is 50 µm. **e** Intracellular citrate (Cit), succinate (Suc), fumarate (Fum) and malate (Mal) levels in osteoclasts from *Phgdh*^*oc+*^ and *Phgdh*^*oc-*^ mice (n = 4; unpaired, two-tailed Student’s *t*-test). Data are means ± s.d. Source data

To further investigate the importance of PSAT1 to generate αKG from glutamate during early osteoclastogenesis, we performed [^13^C_5_/^15^N_2_]glutamine tracing and found that in wild-type osteoclasts, ^15^N incorporation into serine and glycine, which is indicative of PSAT enzyme activity, followed the same dynamic labelling pattern as ^13^C labelling of glutamate and αKG, with a peak in isotopic labelling within the first 3 d of differentiation (Fig. 4a,b and Extended Data Fig. 6a,b). This transient increase in glutamine-derived nitrogen incorporation in serine and glycine mimicked the dynamic pattern of glucose conversion into serine and glycine (Fig. 1e,f). Of note, glutamine uptake was increased during osteoclast differentiation (Extended Data Fig. 6c). To determine the functional role of PSAT1, we treated osteoclast progenitors with the pan-transaminase inhibitor aminooxyacetic acid (AOA) or silenced PSAT1 using shRNA (Extended Data Fig. 7a). Both ways of transaminase inactivation decreased αKG levels, the number of TRAP-positive multinuclear cells (MNCs) and osteoclast-related gene expression (Fig. 4c–i). In line with the early activation of the SSP, inhibition of transaminase activity with AOA only reduced osteoclastogenesis when cells were treated during the first 3 d of differentiation (Fig. 4d–f). Of note, other important αKG-producing transaminases, such as alanine aminotransferase or aspartate aminotransferase 2, did not contribute considerably to αKG production in osteoclasts, nor did they regulate osteoclast differentiation, likely because of the compensatory increase in PSAT1 expression when alanine aminotransferase or aspartate aminotransferase 2 was depleted (Extended Data Fig. 7a–d). Finally, similarly to conditions of *Phgdh* deletion, supplementation of αKG fully restored osteoclast differentiation after PSAT1 silencing (Fig. 4j–l). Taken together, these data indicate that the early stages of osteoclastogenesis depend on induction of the SSP to produce αKG, which is necessary for their differentiation.

### SSP-derived αKG regulates osteoclast differentiation via H3K27 demethylation

**Fig. 5:**
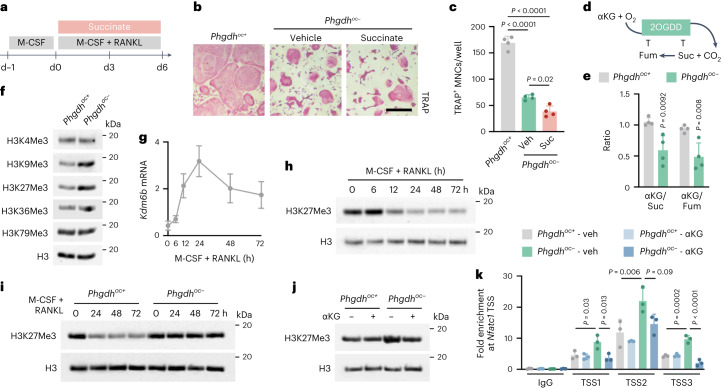
SSP-derived αKG regulates *Nfatc1* transcription by facilitating histone demethylation. **a**, Schematic overview of experimental setup. Osteoclasts were treated with cell-permeable succinate during the entire experiment. **b**,**c**, TRAP staining (**b**) and quantification of TRAP^+^ MNCs (**c**) from *Phgdh*^*oc+*^ and *Phgdh*^*oc*−^ mice treated with either vehicle or succinate (*n* = 4; one-way ANOVA). Scale bar, 50 µm. **d**, Schematic overview of the regulation of 2-oxoglutarate-dependent dioxygenase (2OGDD) activity. Suc, succinate; Fum, fumarate. **e**, Ratio of intracellular αKG to Suc or Fum (*n* = 4; unpaired, two-tailed Student’s *t*-test). **f**, H3K4Me3, H3K9Me3, H3K27Me3, H3K36Me3 and H3K79Me3 protein levels in osteoclasts from *Phgdh*^*oc+*^ and *Phgdh*^*oc*−^ mice. Total H3 levels were used as the loading control. **g**,**h**, *Kdm6b* (encoding for JMJD3) mRNA levels (*n* = 3; **g**) and H3K27Me3 protein levels (**h**) in wild-type osteoclasts at different timepoints during early differentiation. **i**, H3K27Me3 protein levels in osteoclasts from *Phgdh*^*oc+*^ and *Phgdh*^*oc*−^ mice at different timepoints during early differentiation. **j**, H3K27Me3 protein levels in osteoclasts from *Phgdh*^*oc+*^ and *Phgdh*^*oc*−^ mice treated for 72 h with M-CSF/RANKL and supplemented with either vehicle or αKG. **k**, ChIP–qPCR analysis of H3K27Me3 enrichment at different locations in the transcription start site (TSS) of the *Nfatc1* gene locus in osteoclasts from *Phgdh*^*oc+*^ and *Phgdh*^*oc*−^ mice treated either with vehicle or αKG (*n* = 3; two-way ANOVA). Data are means ± s.d. For immunoblots, representative images from three independent experiments are shown. Source data

**Extended Data Fig. 8:**
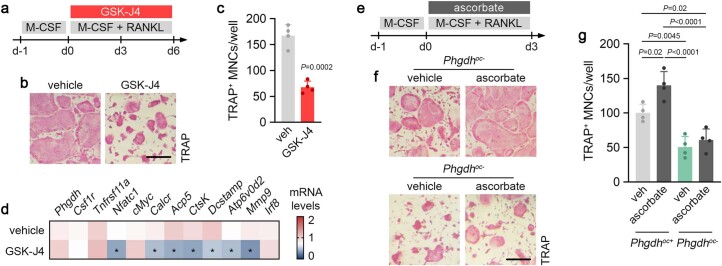
Histone demethylation regulates osteoclast differentiation. **a** Schematic overview of experimental setup. Osteoclasts were treated with the JMJD3 inhibitor GSK-J4 during the entire experiment. **b**, **c** TRAP staining (**b**) and quantification of TRAP^+^ MNCs (**c**) from wild-type mice treated with either vehicle or GSK-J4 (n = 4; unpaired, two-tailed Student’s *t*-test). **d** *Phgdh*, *Csf1r*, *Tnfrsf11a* (encoding for RANK), *Nfatc1* (*P* = 0.007), *cMyc*, *Calcr* (*P* = 0.015), *Acp5* (*P* = 0.007), *Ctsk* (*P* = 0.008), *Dcstamp* (*P* = 0.02), *Atp6v0d2* (*P* = 0.04), *Mmp9* (*P* < 0.0001) and *Irf8* mRNA levels in osteoclasts treated with either vehicle or GSK-J4 (n = 4; unpaired, two-tailed Student’s *t*-test with *p < 0.05 vs vehicle). **e** Schematic overview of experimental setup. Osteoclasts were treated with ascorbate during early osteoclast differentiation (that is from d0 to d3). **f-g** TRAP staining (**f**) and quantification of TRAP^+^ MNCs (**g**) from *Phgdh*^*oc+*^ and *Phgdh*^*oc-*^ mice treated with vehicle or ascorbate (n = 4; two-way ANOVA). Scale bar is 50 µm. Data are means ± s.d. Source dat

We next investigated how αKG levels control osteoclastogenesis. Several observations suggested that the contribution of αKG to TCA cycle anaplerosis is likely not important for osteoclastogenesis. First, [^13^C_5_]glutamine contributed substantially to glutamate and αKG, but much less to other TCA cycle metabolites in wild-type cells and this contribution did not show a transient increase during early osteoclastogenesis (Extended Data Fig. 6b,d). Second, *Phgdh* deletion reduced αKG levels by ~50% (Fig. 3i), but only marginally affected the levels of TCA cycle intermediates (~10% to 15%; Extended Data Fig. 7e). Finally, administration of cell-permeable succinate did not rescue the impaired osteoclastogenesis caused by PHGDH inactivation (Fig. 5a–c). In addition to serving as a substrate for the TCA cycle, αKG is also a co-substrate for a large family of dioxygenases that convert αKG to succinate as part of the enzymatic reaction^28^ (Fig. 5d). Metabolic regulation of αKG-dependent dioxygenases, including the Jumonji-domain (Jmj) family of histone demethylases, has been implicated in chromatin regulation, gene expression and cell fate decisions^28^, but how metabolic adaptations regulate osteoclast epigenetics and thus their differentiation is still poorly understood. We observed that the αKG/succinate and αKG/fumarate ratio was decreased in PHGDH-deficient osteoclasts by almost 50% (Fig. 5e), which can compromise the activity of the dioxygenases^28^. In line, trimethylated histone 3 lysine 9 (H3K9Me3) and H3K27Me3 levels were higher in mutant cells, whereas H3K4Me3, H3K36Me3 and H3K79Me3 levels were not affected, suggesting that SSP-derived αKG favours the demethylation of specific histone marks (Fig. 5f). In osteoclasts, H3K27Me3 functions as a repressing epigenetic mark and blocks terminal differentiation^29^. We noticed that in wild-type cells, RANKL treatment rapidly induced KDM1 lysine (K)-specific demethylase 6B (*Kdm6b*, encoding for JMJD3) gene expression in a similar fashion as the activation of genes of the SSP, of which PSAT1 produces αKG, the co-substrate for JMJD3 activity (Figs. 1j,k and 5g). Accordingly, treatment with a small-molecule JMJD3 inhibitor decreased osteoclast differentiation and osteoclast-related gene expression (Extended Data Fig. 8a–d). Furthermore, the RANKL-induced time-dependent decrease in H3K27Me3 levels was only observed in wild-type osteoclasts and not in PHGDH-deficient cells, but simultaneous treatment of mutant cells with αKG decreased H3K27Me3 levels, whereas it had no effect in wild-type osteoclasts (Fig. 5h–j). Further evidence that impaired αKG synthesis is the limiting factor for histone demethylation in PHGDH knockout cells is the observation that supplementation with ascorbic acid, which normally stimulates JMJD3 activity and thus reduces H3K27 trimethylation^29^, did not rescue the differentiation defect (Extended Data Fig. 8e–g).

It is known that H3K27Me3 represses osteoclastogenesis by negatively regulating the expression of the pro-osteoclastogenic transcription factor NFATc1 (ref. ^29^) and we hypothesized that the high H3K27Me3 levels that result from loss of PHGDH poise *Nfatc1* transcription. Using chromatin immunoprecipitation with quantitative PCR (ChIP–qPCR), we indeed found that trimethylation of H3K27 near the transcription start site of the *Nfatc1* gene locus was considerably increased in mutant osteoclasts, which was rescued after supplementation with cell-permeable αKG (Fig. 5k). Taken together, our data indicate that SSP-derived αKG is required to support JMJD3-mediated H3K27 demethylation at the *Nfatc1* locus, thereby facilitating *Nfatc1* transcription and consequent osteoclast differentiation.

### PHGDH inhibition protects mice from osteoporotic bone loss

**Fig. 6:**
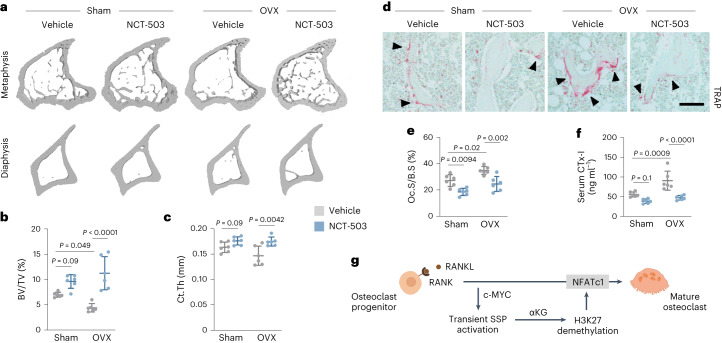
Pharmacological PHGDH inhibition prevents osteoporotic bone loss in mice. **a**–**c**, 3D micro-CT models (**a**) of the tibial metaphysis (top) and diaphysis (bottom) with quantification of trabecular bone volume (BV/TV; **b**) and cortical thickness (Ct.Th; **c**) in sham-operated or ovariectomized (OVX) mice, treated with either vehicle or NCT-503 (*n* = 6; two-way ANOVA). **d**,**e**, TRAP staining of the tibial metaphysis (**d**) with quantification (**e**) of osteoclast surface per bone surface (Oc.S/B.S) in sham-operated or OVX mice, treated with either vehicle or NCT-503 (*n* = 6; two-way ANOVA). Scale bar, 50 µm. **f**, Serum CTx-I levels in sham-operated or OVX mice, treated with either vehicle or NCT-503 (*n* = 6; two-way ANOVA). **g**, Schematic overview of research findings. A transient, RANKL-induced activation of the SSP is required to generate αKG during early osteoclastogenesis, which is subsequently used by histone demethylases to remove the repressive histone mark H3K27Me3 at the *Nfatc1* gene locus, thereby inducing NFATc1 expression and consequent osteoclast maturation. Created with BioRender.com. Data are means ± s.d. Source data

**Extended Data Fig. 9:**
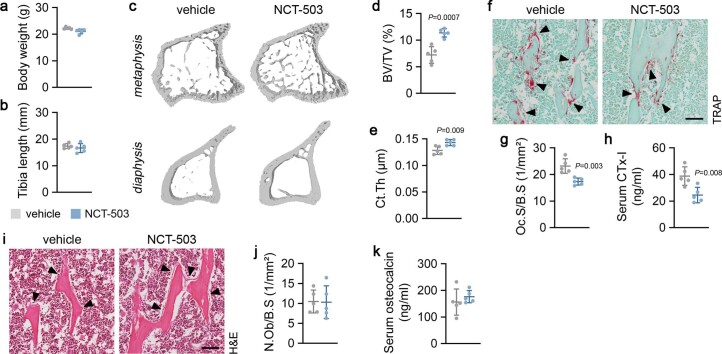
Systemic administration of NCT-503 increases bone mass. **a**, **b** Body weight (**a**) and tibia length (**b**) of male mice treated with either vehicle or NCT-503 (n = 5). **c-e** 3D microCT models (**c**) of the tibial metaphysis (top) and diaphysis (bottom) with quantification of trabecular bone volume (BV/TV; **d**) and cortical thickness (Ct.Th; **e**) in vehicle and NCT-503-treated mice (n = 5; unpaired, two-tailed Student’s *t*-test). **f-g** TRAP staining of the tibial metaphysis (**f**) with quantification (**g**) of osteoclast surface per bone surface (Oc.S/B.S) in vehicle and NCT-503-treated mice (n = 5; unpaired, two-tailed Student’s *t*-test). Scale bar is 100 µm. **h** Serum CTx-I levels in vehicle and NCT-503-treated mice (n = 5; unpaired, two-tailed Student’s *t*-test). **i-j** H&E staining of the tibial metaphysis (**i**) with quantification of the number of osteoblasts per bone surface (N.Ob/B.S;) in vehicle and NCT-503-treated mice (n = 5). Scale bar is 100 µm. **k** Serum osteocalcin levels in vehicle and NCT-503-treated mice (n = 5). Data are means ± s.d. Source data

**Extended Data Fig. 10:**
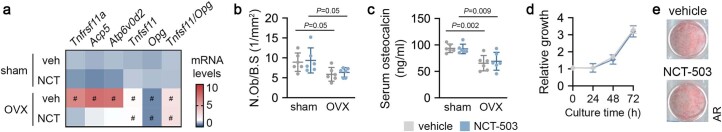
NCT-503 treatment does not affect osteoblast properties. **a** *Tnfrsf11a* (encoding for RANK; OVX-veh: *P* < 0.0001), *Acp5* (OVX-veh: *P* < 0.0001), *Atp6v0d2* (OVX-veh: *P* < 0.0001), *Tnfsf11* (encoding for RANKL; OVX-veh: *P* < 0.0001; OVX-NCT: *P* < 0.0001), *Opg* (OVX-veh: *P* = 0.03; OVX-NCT: *P* = 0.03) and *Tnfsf11/Opg* mRNA levels (OVX-veh: *P* = 0.002; OVX-NCT: *P* = 0.001) in total bone from sham-operated or OVX mice, treated with either vehicle (veh) or NCT-503 (NCT) (n = 6; two-way ANOVA with ^#^p < 0.05 vs sham-veh). **b-c** Quantification of the number of osteoblasts per bone surface (N.Ob/B.S; **b**) and serum osteocalcin levels (**c**) in sham-operated or ovariectomized (OVX) mice, treated with vehicle or NCT-503 (n = 6; two-way ANOVA). **d**, **e** Growth curve of osteoprogenitors (**d**) and Alizarin red (AR) staining of differentiated osteoblasts (**e**), treated with vehicle or NCT-503 (n = 3). Data are means ± s.d. Source data

Finally, we questioned whether we could exploit the therapeutic potential of blocking osteoclastogenesis, by pharmacologically reducing PHGDH activity by NCT-503. This approach has the same in vivo effect on bone as osteoclast-specific PHGDH deletion (Extended Data Fig. 9a–k). We used a preclinical oestrogen deficiency-induced bone loss model, mimicking postmenopausal osteoporosis, in which the adverse effects of oestrogen deficiency on skeletal properties are mainly caused by an osteoclast-driven response^30^. We performed ovariectomy (OVX) or sham operation in 10-week-old female mice and treated them for 5 weeks intraperitoneally with NCT-503. OVX resulted in a strong reduction in bone mass (BV/TV: −37%; Ct.Th: −12.5%) in vehicle-treated mice, and this bone loss was completely prevented by NCT-503 treatment (Fig. 6a–c). As expected, we observed an increase in osteoclast number and activity in ovariectomized control mice (Fig. 6d–f and Extended Data Fig. 10a). In contrast, NCT-503 administration fully blunted the increase in osteoclast-mediated bone resorption without affecting osteoblast number or activity (Fig. 6d–g and Extended Data Fig. 10b–e), indicating that pharmacological PHGDH inhibition may be a valuable therapeutic strategy to block osteoporotic bone loss.

## Discussion

Osteoclast differentiation depends on the combined action of M-CSF and RANKL, which stimulate several signalling pathways that converge to induce the expression and function of the key transcriptional regulator NFATc1 (refs. ^6,7^). Recent studies suggest that metabolic pathways are changed during osteoclast formation and function^9,10,16,31^, but whether and how specific metabolites are critical for the differentiation process remains poorly understood. In this study, using metabolomic analyses and transgenic mouse models, we demonstrate that RANKL-induced activation of the SSP is required during early osteoclastogenesis, not to maintain intracellular serine levels, but to generate αKG, which epigenetically regulates *Nfatc1* expression and thus drives osteoclast differentiation (Fig. 6g).

Recent studies, predominantly in tumour cells but also in noncancerous cell types including skeletal cells, have identified specific metabolites as key drivers of cell phenotype and function^8,12–14,32^. However, data on the causal relationship between metabolic pathways and osteoclastogenesis are still limited. During osteoclast differentiation, mitochondrial respiration increases, likely to accommodate the bioenergetic demands of bone resorption, whereas elevated glycolysis may contribute to local acidification that dissolves bone minerals^9,10^. Our study now evidently shows that the production of specific metabolites is required to initiate the early phases of osteoclast differentiation. More precisely, timely αKG synthesis is necessary to stimulate the expression of NFATc1 by epigenetic mechanisms, which consequently induces the typical osteoclast-related gene expression profile. αKG can be produced by several metabolic pathways^28^, but osteoclastogenesis depends on a transient activation of the SSP for the generation of αKG. Our molecular and metabolic findings show that during early osteoclastogenesis the expression of the SSP enzymes PHGDH and PSAT1 is increased, resulting in enhanced glucose carbon flux to serine, which is accompanied by temporarily increased glutamine-derived carbon and nitrogen incorporation into αKG and serine, respectively. Importantly, activation of the SSP precedes the peak in NFATc1 expression and its physiological significance is further underscored by the fact that inhibition of the SSP enzymes PHGDH or PSAT1 genetically or pharmacologically reduces NFATc1 expression and osteoclastogenesis, defects that can be rescued by adding αKG. This increase in SSP activity is functionally important, as in vivo inactivation of PHGDH in osteoclast precursors results in increased bone mass.

A major role of αKG is to regulate the activity of enzymes involved in epigenetic regulation of gene expression, such as DNA and histone demethylases. While Dnmt3a-mediated DNA methylation is important for osteoclast functioning by regulating the expression of anti-osteoclastogenic genes^20^, our study uncovers a critical role for αKG-mediated histone demethylation. In osteoclast precursors, H3K27 trimethylation poises *Nfatc1* transcription, but during RANKL-mediated differentiation, repressive H3K27Me3 marks are removed by the histone demethylase JMJD3, thereby inducing *Nfatc1* expression and consequently osteoclast maturation^29^. We now show that αKG generated by the SSP is required for the JMJD3-mediated H3K27Me3 demethylation to induce *Nfatc1* transcription. These findings suggest that the increased glycolytic flux observed during early osteoclastogenesis is, at least partially, required to fuel the SSP that generates αKG for histone demethylase activity. Two recent studies suggest that supplementation of αKG can inhibit osteoclastogenesis^33,34^, although these inhibitory effects are elicited at concentrations that are at least twofold higher than the 0.25 mM concentration that was used in this study. These high concentrations may also affect the enzymatic activity of other αKG-dependent dioxygenases^28^, such as the HIF-prolyl hydroxylases as observed in ref. ^34^, which can influence cell behaviour differently.

At the molecular level, we found that RANKL treatment increases the expression of SSP-related enzymes through c-MYC, which also regulates other metabolic pathways during osteoclastogenesis. Indeed, c-MYC also induces oxidative phosphorylation^27^ and the produced energy may be used to generate *S*-adenosyl methionine to support Dnmt3a-mediated DNA methylation^20^, although the in vivo significance of increased oxidative phosphorylation has not been formally tested. Moreover, whether and how other metabolic pathways converge in the temporal and dynamic regulation of the epigenome during osteoclast differentiation is an interesting question that requires further research.

Taken together, this study reveals a RANKL-induced metabolic–epigenetic coupling mechanism during osteoclast differentiation whereby αKG generation via the SSP is linked to histone demethylation and *Nfatc1* transcription. Our work has also important translational and therapeutic implications, as treatment of mice with a PHGDH small-molecule inhibitor completely prevented OVX-induced bone loss without affecting osteoblast parameters or inducing overall toxicity^35,36^, suggesting that the SSP represents a new therapeutic target to treat osteoporosis.

## Methods

### Animal studies

#### Transgenic mice

Osteoclast-specific deletion of PHGDH was obtained by crossing *Phgdh*^*lox/lox*^ mice (in which *Phgdh* exons 4 and 5 are flanked by LoxP sites^37^) with *lysozyme 2* gene promoter-Cre (*Lyz2*-Cre) transgenic mice^38^ (resulting in *Lyz2*-Cre^+^;*Phgdh*^*lox/lox*^ mice, referred to as *Phgdh*^*oc*−^ mice). *Lyz2*-Cre^−^;*Phgdh*^*lox/lox*^ (*Phgdh*^*oc+*^) littermates were used as controls in all experiments. Mouse phenotyping was performed on 8-week-old male and female mice. All mice (100% C57BL/6J background) were housed and bred under conventional conditions (that is, at 18–23 °C and 40–60% humidity with a 12-h light–dark cycle; Proefdierencentrum Leuven). All experimental procedures were approved by the Institutional Animal Care and Research Advisory Committee of the KU Leuven (protocol no. P140/2020).

#### OVX

OVX was performed in 10-week-old female C57BL/6 mice, as described before^39^. In sham-operated mice, the ovaries were exposed but left intact. For PHGDH inhibition, mice were injected intraperitoneally with NCT-503 (40 µg per gram of body weight) every other day, starting 1 d after surgery. NCT-503 was dissolved in a vehicle composed of 5% ethanol and 35% PEG300 in a 30% hydroxypropyl-β-cyclodextrin solution^40^. All chemicals were from Sigma-Aldrich. Five weeks after surgery, tibiae and serum were collected and processed as described below.

### X-ray micro-CT

Micro-CT analysis of mineralized bone mass in tibiae was performed using the SkyScan 1272 system (Bruker) at a pixel size of 5 µm with 50 kV tube voltage, 200 µA current and 0.5 mm aluminium filter^39,41^. Projection data were reconstructed using the NRecon software (Bruker) and trabecular and cortical volumes of interest were selected manually. For trabecular bone, the region between 0.5 and 3.5 mm distal from the growth plate was selected, and for cortical bone, the region between 2 and 2.5 mm was selected. 3D morphometric parameters were calculated using the CT Analyzer software (Bruker) at a threshold index of 100 to 255 (global Otsu thresholding). Data were presented according to the guidelines of the American Society for Bone and Mineral Research^42^. 3D image rendering was performed using the 3D visualization software (Bruker).

### Serum biochemistry

Serum osteocalcin was measured by an in-house radioimmunoassay^39^ and serum CTx-I levels were measured by a RatLaps ELISA kit (Immunodiagnostic Systems).

### (Immuno)histochemistry and histomorphometry

Histomorphometric analyses on mouse tibiae were performed as previously described^39,41,43^. Briefly, osteoclasts were visualized and quantified on TRAP-stained sections. Osteoblast number was quantified using H&E-stained sections, and unmineralized (osteoid) or mineralized bone matrix was quantified on Goldner or Von Kossa-stained sections, respectively. To analyse dynamic bone parameters, calcein (16 mg per kg body weight; Sigma-Aldrich) was administered via intraperitoneal injection 4 d and 1 d before euthanasia.

For PHGDH immunohistochemical staining, bone sections were incubated overnight with anti-PHGDH (66350, Cell Signaling Technology). A goat-anti-rabbit Alexa Fluor 488-labelled secondary antibody was used for signal visualization, and sections were stained with Hoechst to visualize cell nuclei.

Images were taken using an Axioplan 2 microscope (Zeiss), and histomorphometric analysis was performed using related Axiovision software (Zeiss). Measurements were performed on at least three sections, each at least 40 µm apart. For each section, three regions of interest were selected starting at 150 µm from the distal end of the growth plate. Data were expressed according to the American Society for Bone and Mineral Research standardized histomorphometry nomenclature^44^.

### Cell isolation and culture

#### Isolation and culture

Primary osteoclast precursors were isolated from long bones of 8-week-old male and female mice^39^. Briefly, bones were dissected, and muscle and connective tissue were removed using sterile gauze. Epiphyses were discarded, and bone marrow was flushed using growth medium (glutaMAX-1 αMEM, supplemented with 100 units per millilitre penicillin, 50 µg ml^−1^ streptomycin and 10% FBS; all from Gibco). Bone marrow-derived cells were collected after Ficoll-Paque PLUS (Cytiva) density gradient centrifugation and cultured with 10 ng ml^−1^ M-CSF (R&D Systems) in growth medium for 24 h. Non-adherent cells were collected and plated in growth medium supplemented with 20 ng ml^−1^ M-CSF and 100 ng ml^−1^ RANKL (PeproTech) to induce osteoclast differentiation. Medium was refreshed at day 3 of differentiation unless otherwise specified.

Primary chondrocytes or skeletal stem/progenitor cells (SSPCs) were isolated from long bones of 3- to 5-day-old mice or 7- to 9-week-old male mice, respectively, as described before^43,45,46^. After isolation, cells were passed through a 70-μm-pore cell strainer, washed and plated in growth medium at a density of 5 × 10^3^ cells per cm². RNA was isolated after 3 d of culture, as described below. For SSPC growth curve, DNA was isolated and DNA content was quantified using Hoechst incorporation. For osteogenic differentiation, SSPCs were seeded in growth medium at a density of 2.5 × 10^3^ cells per cm^2^. Following confluency, growth medium was switched to osteogenic differentiation medium (growth medium containing 50 µg ml^−1^ l-ascorbic acid and 10 mM β-glycerophosphate; both from Sigma-Aldrich). Osteogenic differentiation medium was refreshed twice per week and mineralization was visualized after staining with 0.1% Alizarin Red (Sigma-Aldrich; pH 6.2) at day 21 of differentiation.

#### In vitro treatments

When indicated, the following compounds or metabolites were used during osteoclastogenesis: Me-Ser-ester (1.2 mM), dimethyl-αKG (0.25 mM), formate (1 mM), AOA (0.5 mM), dimethyl-succinate (4 mM), NCT-503 (5 µM), GSK-J4 (10 µM) or ascorbate (0.5 mM), or cultured in five times the normal serine concentration in the culture medium (that is, final concentration of 1.2 mM). Treatment regimens are specified in the figures and respective figure legends. Dimethylsulfoxide was used as the vehicle control. All compounds were acquired from Sigma-Aldrich.

### TRAP staining

To assess osteoclast formation, we performed in vitro TRAP staining on day 6 of differentiation unless otherwise indicated^47^. Briefly, cells were washed with PBS, fixed (4% paraformaldehyde for 10 min at room temperature) and permeabilized (0.5% Triton X-100 for 5 min at room temperature) before adding TRAP staining solution (0.1 mg ml^−1^ naphthol AS-MX phosphate, 10 µl ml^−1^ *N*,*N*-dimethylformamide, 0.6 mg ml^−1^ fast red violet LB salt in 0.1 M sodium acetate buffer at pH 5.0). TRAP-positive cells with more than three nuclei were counted as osteoclasts.

### Genetic targeting

For gene knockdown, M-CSF-treated osteoclast precursors were transduced with a lentivirus carrying c-MYC shRNA (TRCN0000234925; Sigma-Aldrich) or PSAT1 shRNA (TRCN0000120419; Sigma-Aldrich) in the presence of 8 µg ml^−1^ polybrene. After 24 h, cells were collected and treated with M-CSF and RANKL to assess osteoclast formation or metabolites. An empty vector was used as the control.

### Mass spectrometry-based metabolomics

For metabolic tracing experiments, wild-type cells were cultured in the presence of 5 mM [^13^C_6_]glucose or 2 mM [^13^C_5_/^15^N_2_]glutamine (Cambridge Isotope Laboratories) during 3 d in the following conditions: from day 0 to day 3 in M-CSF-treated cells, from day 0 to day 3 in M-CSF/RANKL-treated cells and from day 3 to day 6 in M-CSF/RANKL-treated cells. Intracellular metabolites of wild-type and PHGDH-deficient osteoclasts were quantified on day 3 of differentiation unless specified otherwise. To isolate metabolites, cells were washed with ice-cold 0.9% saline and scraped in 80% methanol supplemented with d27 myristic acid. Samples were analysed using liquid chromatography–mass spectrometry as described before^32,40^ and metabolite annotation was performed using an in-house library (Metabolomics Core Facility, KU Leuven). Metabolites of interest were analysed using Xcalibur software (Thermo Fisher Scientific) and raw metabolite peak values were normalized to protein content.

### Gene and protein expression analysis

#### Gene expression

RNA was extracted from cells or tissues using the NucleoSpin RNA Isolation Kit (Macherey Nagel), and mRNA was reverse transcribed to cDNA using the Superscript II Reverse transcriptase (Thermo Fisher Scientific). Gene expression analysis was performed using specific oligonucleotide primers (Supplementary Table 2). Expression values were calculated using the 2^−ΔΔCt^ method and normalized to *Hprt* expression (StepOne Real-Time PCR software).

#### Protein expression

Total cell lysates and nuclear protein-enriched lysates were collected using appropriate cell lysis buffers, and western blot analysis was performed as described previously^40,48^. Analysis of histone methylation was performed 24 h after induction of osteoclastogenesis, unless otherwise specified. Proteins were separated by SDS–PAGE, transferred to nitrocellulose membranes and incubated overnight with primary antibodies against β-actin (A5441, Sigma-Aldrich), c-MYC (5605, Cell Signaling Technology), H3K4Me3 (9751, Cell Signaling Technology), H3K9Me3 (13969, Cell Signaling Technology), H3K27Me3 (9733, Cell Signaling Technology), H3K36Me3 (4909, Cell Signaling Technology), H3K79Me3 (74073, Cell Signaling Technologies), lamin A/C (sc-376248, Santa Cruz Biotechnologies), NFATc1 (sc-7294, Santa Cruz Biotechnologies), PHGDH (66350, Cell Signaling Technologies) or PSAT1 (NBP1-55368, Bio-Techne). Appropriate anti-mouse or anti-rabbit horseradish peroxidase-conjugated secondary antibodies were used for chemiluminescent protein detection (Western Lightning Plus, PerkinElmer).

### Proliferation analysis

Cell proliferation was measured by BrdU incorporation using the Cell Proliferation Biotrack ELISA system (Cytiva) according to the manufacturer’s instructions. Briefly, 24 h after seeding, M-CSF-treated osteoclast progenitors were cultured in the presence of BrdU for 4 h before measurement, and the obtained values were normalized to DNA content.

### Survival analysis

Osteoclast survival was analysed using the Caspase-3 Activity Assay Kit (Cell Signaling Technology) according to the manufacturer’s instructions. Analysis was performed after 6 d of M-CSF/RANKL treatment and the obtained values were normalized to DNA content.

### Analysis of myeloid cell populations

Bone marrow myeloid cells were obtained by flushing tibiae and femurs with growth medium, and enriched for CD45 expression using CD45 MicroBeads (Miltenyi Biotec) and magnetic-activated cell sorting according to the manufacturer’s instructions. Cells were spun down and labelled with the following antibodies for 45 min at 4 °C: APC-CD11b (101211, BioLegend), FITC-Gr-1 (108405, BioLegend), FITC-F4/80 (123107, BioLegend), PE-CD115 (165203, BioLegend) and PerCP-CD19 (115531, BioLegend). The number of Gr-1^+^CD11b^+^ neutrophils, F4/80^+^CD11b^+^ macrophages, CD115^+^CD11b^+^ monocytes and CD19^+^ B cells was assessed by flow cytometry (BD FACSCanto, BD Biosciences) as described before^49^ and quantified using Kaluza software (Beckman Coulter).

Circulating myeloid cells in blood were analysed using the Sysmex XN1000 and XN2000 Hematology Analyzer (Sysmex Corporation; University Hospital Leuven).

### ChIP–qPCR

When osteoclast precursors were treated for 72 h, they were fixed using 1% formaldehyde, washed and collected by centrifugation (1,000*g* for 5 min at 4 °C). The pellet was resuspended in RIPA buffer (50 mM Tris-HCl pH 8, 150 mM NaCl, 2 mM EDTA, 1% Triton X-100, 0.5% sodium deoxycholate, 1% SDS and 1% protease inhibitors), homogenized, incubated on ice for 10 min and sonicated. The samples were centrifuged (16,000*g* for 10 min at 4 °C) and from the supernatant, shared chromatin was used as input and incubated with an anti-H3K27Me3 antibody (9733, Cell Signaling Technology). Rabbit IgG (2729, Cell Signaling Technology) was used as the isotype control. After pull-down using Pierce Protein A/G Magnetic Beads (Thermo Fisher Scientific), followed by RNA and protein digestion, DNA was purified using Agencourt AMPure XP (Beckman Coulter) according to the manufacturer’s instructions. RT–qPCR was performed using SYBR GreenER qPCR SuperMix Universal (Thermo Fisher Scientific) and specific primers to detect H3K27Me3 enrichment in the transcription start site of *Nfatc1* (set 1: Fw 5′-CAGCGACATGAAAGGAACAATC-3′, Rev 5′-GGACACCTGGCTCATCTTTAG-3′; set 2: Fw 5′-GGCAGAACTCTTGTCTGGATAC-3′, Rev 5′-GCCTTAGCTGCTTCTCACTAAA-3′; set 3: Fw 5′-GGTCAAGTTATCCCTGCTGAA-3′, Rev 5′-ACAATGACATGACCCAGACC-3′).

### Statistics

Data are presented as means ± s.d., and *n* values represent the number of independent experiments performed or the number of individual mice phenotyped. Cells/mice were randomly assigned to the different experimental groups. For each independent in vitro experiment, at least three technical replicates were used. For immunoblots, at least three independent experiments were performed, and representative images are shown. No statistical methods were used to predetermine sample sizes, but our sample sizes are similar to those reported in previous publications^40,45,48^. Data distribution was assumed to be normal, but this was not formally tested. Data collection and analysis were not performed blind to the conditions of the experiments. Statistical analysis (GraphPad Prism 9 software) was performed using the unpaired two-tailed Student’s *t*-test or one-way/two-way ANOVA followed by the Tukey–Kramer post hoc test, as specified in the figure legends. *P* values < 0.05 were considered statistically significant.

### Reporting summary

Further information on research design is available in the Nature Portfolio Reporting Summary linked to this article.

### Supplementary information

Supplementary Information Supplementary Tables 1 and 2.

Reporting Summary

Supplementary Data Source data for Supplementary Table 1.

### Source data

Source Data Fig. 1 Statistical source data.

Source Data Fig. 2 Statistical source data.

Source Data Fig. 3 Statistical source data.

Source Data Fig. 4 Statistical source data.

Source Data Fig. 5 Statistical source data.

Source Data Fig. 6 Statistical source data.

Source Data Extended Data Fig. 1 Statistical source data.

Source Data Extended Data Fig. 2 Statistical source data.

Source Data Extended Data Fig. 3 Statistical source data.

Source Data Extended Data Fig. 4 Statistical source data.

Source Data Extended Data Fig. 5 Statistical source data.

Source Data Extended Data Fig. 6 Statistical source data.

Source Data Extended Data Fig. 7 Statistical source data.

Source Data Extended Data Fig. 8 Statistical source data.

Source Data Extended Data Fig. 9 Statistical source data.

Source Data Extended Data Fig. 10 Statistical source data.

Source Data—uncropped gels Uncropped western blots of both main figures and Extended Data figures.

## Data Availability

Data will be made available from the corresponding author upon reasonable request. Source data are provided with this paper.
