# Supplementary material for: The serine synthesis pathway drives osteoclast differentiation through epigenetic regulation of NFATc1 expression
Source: Nat Metab. 2024 Jan 10;6(1):141–52. doi: 10.1038/s42255-023-00948-y (PMC10822776; doi:10.1038/s42255-023-00948-y)
Supplement: Supplementary file 1 — Supplementary Tables 1 and 2. [file 42255_2023_948_MOESM1_ESM.pdf]

nature metabolism

Article https://doi.org/10.1038/s42255-023-00948-y

The serine synthesis pathway drives osteoclast differentiation through epigenetic regulation of NFATc1 expression

In the format provided by the authors and unedited

Supplementary Data Table 1. Phenotype of 8-week-old female *Phgdh*$^{oc+}$ and *Phgdh*$^{oc-}$ mice.

	Phgdh^{oc+}	*Phgdh*$^{oc-}$
Body weight (g)	19.3 ± 2.3	17.8 ± 0.78
Tibia length (mm)	15.2 ± 1.8	14.2 ± 1.4
BV/TV (%)	6.9 ± 1.6	11.2 ± 2.4 *
Tb.Th (μm)	40.5 ± 2.2	44.3 ± 3.0 *
Tb.N (1/μm)	0.0024 ± 0.0014	0.0034 ± 0.0021
Tb.Sp (μm)	289.5 ± 40.6	237.6 ± 50.1
Ct.Th (μm)	121.0 ± 10.1	136.5 ± 9.0 *
Ec.Pm (mm)	5.9 ± 0.92	5.2 ± 0.12 *
Ma.Ar (mm^2)	0.98 ± 0.11	0.82 ± 0.14 *
Ps.Pm (mm)	6.4 ± 0.77	6.1 ± 0.27
Po (%)	2.5 ± 0.58	2.3 ± 0.90

BV/TV: P=0.0018; Tb.Th: P=0.020; Ct.Th: P=0.008; Ec.Pm: P=0.03; Ma.Ar: P=0.042 (unpaired, two-tailed Student's t-test with *p<0.05 vs *Phgdh*$^{oc+}$).

Supplementary Data Table 2. Primer sequences for qRT-PCR.

Gene	Forward (5'-3')	Reverse (5'-3')
Acp5	ACTTCCCCAGCCCTTACTACC	TCAGCACATAGCCCACACCG
Atp6v0d2	TCAGATCTCTTCAAGGCTGTGCTG	GTGCCAAATGAGTTCAGAGTGATG
Bglap	GGCCCTGAGTCTGACAAAGC	GCTCGTCACAAGCAGGGTTAA
CalcRec	AACCGAACCTGGTCCAACTATACT	TCAGCATGGAAGCAACCAAA
cMyc	GATTCCACGGCCTTCTCTC	TCTTCTTGCTCTTCTTCAGAGTC
Col1a1	TGTCCCAACCCCCAAAGAC	CCCTCGACTCCTACATCTTCTGA
Csfr1	GCAGTACCACCATCCACTTGTA	GTGAGACACTGTCCTTCAGTGC
Ctsk	TGTGGACTGTGTGACTGAGAATTATG	CCTTTGCCGTGGCGTTAT
Dcstamp	AAACTACGTGGAGAGAAGCAAGGA	CAGAATAATACTGAGAGGAACCCAAGT
Got2	TGAAGTGTTGAAAAGCGGCCG	TTGGGCAGAAAGACATCTCGG
Gpt	CTGAGGTTATCCGTGCCAATA	CCGGACTGCTCAGAAGATTG
Hprt	TTATCAGACTGAAGAGCTACTGTAATGATC	TTACCAGTGTCAATTATATCTTCAACAATC
Irf8	GGAAAGCCTTACCTGCTGAC	AAGGTCACCGTGGTCCTTAG
Jmjd3	CAACTCCATCTGGCTGTTACTG	CCTTCTGCAACCAATTCCAG
Mmp9	CCAAAGACCTGAAAACCTCCAA	GTAGAGACTGCTTCTCTCCCATCAT
Nfatc1	CTCGAAAGACAGCACTGGAGCAT	CGGCTGCCTTCCGTCTCATAG
Opg	GAAGGGCGTTACCTGGAGATC	CTGAATTAGCAGGAGGCCAAAT
Phgdh	CGATGAAAGATGGCAAATGGG	GCCACCTCTCTTCCAATTCT
Psat1	GCAGCTACTAGACTACAGAGGA	GCTAGCAATTCCCTCACAAGA
Rank	GAGCTCAGCATCCCTTGCA	CCCTGGTGTGCTTCTAGCTTTC
Rankl	CATTTGCACACCTCACCATCA	TTGCTTAACGTCATGTTAGAGATCTTG
Runx2	TACCAGCCACCGAGACCAA	AGAGGCTGTTTGACGCCATAG
Slc1a4	CAGTGGACTCTTTCCTCGATTT	ATGGGTCACCACTGTGTAATC
Slc6a9	GGGAGGAGCCTTCATGTTC	AGGAAAGCTCCATGAAGAGAG
