# Supplementary material for: The serine synthesis pathway drives osteoclast differentiation through epigenetic regulation of NFATc1 expression
Source: Nat Metab. 2024 Jan 10;6(1):141–52. doi: 10.1038/s42255-023-00948-y (PMC10822776; doi:10.1038/s42255-023-00948-y)
Supplement: Supplementary file 2 — Reporting Summary [file 42255_2023_948_MOESM2_ESM.pdf]

nature portfolio

Corresponding author(s): Geert Carmeliet

Last updated by author(s): Oct 30, 2023

Reporting Summary

Nature Portfolio wishes to improve the reproducibility of the work that we publish. This form provides structure for consistency and transparency in reporting. For further information on Nature Portfolio policies, see our Editorial Policies and the Editorial Policy Checklist.

Statistics

For all statistical analyses, confirm that the following items are present in the figure legend, table legend, main text, or Methods section.

n/a	Confirmed	
☐	☒	The exact sample size (*n*) for each experimental group/condition, given as a discrete number and unit of measurement
☐	☒	A statement on whether measurements were taken from distinct samples or whether the same sample was measured repeatedly
☐	☒	The statistical test(s) used AND whether they are one- or two-sided<br>*Only common tests should be described solely by name; describe more complex techniques in the Methods section.*
☒	☐	A description of all covariates tested
☐	☒	A description of any assumptions or corrections, such as tests of normality and adjustment for multiple comparisons
☐	☒	A full description of the statistical parameters including central tendency (e.g. means) or other basic estimates (e.g. regression coefficient) AND variation (e.g. standard deviation) or associated estimates of uncertainty (e.g. confidence intervals)
☐	☒	For null hypothesis testing, the test statistic (e.g. *F*, *t*, *r*) with confidence intervals, effect sizes, degrees of freedom and *P* value noted<br>*Give P values as exact values whenever suitable.*
☒	☐	For Bayesian analysis, information on the choice of priors and Markov chain Monte Carlo settings
☒	☐	For hierarchical and complex designs, identification of the appropriate level for tests and full reporting of outcomes
☒	☐	Estimates of effect sizes (e.g. Cohen's *d*, Pearson's *r*), indicating how they were calculated

Our web collection on statistics for biologists contains articles on many of the points above.

Software and code

Policy information about availability of computer code

Data collection	qRT-PCR: StepOne Real-Time PCR software v2.3<br>13C tracing: Xcalibur v4.3<br>imaging: Zeiss AxioVision 4.9.1<br>statistics: GraphPad Prism 9<br>microCT: SkyScan 1272 Control Software v1.5<br>flow cytometry: BD FACSCanto II system software v3.0
Data analysis	microCT: CT Analyzer 1.16.4.1, 3D Visualization software v2.3.2.0<br>statistics: GraphPad Prism 9<br>graphs: GraphPad Prism 9<br>image analysis: Zeiss AxioVision 4.9.1<br>flow cytometry: Kaluza 2.2.1 software

For manuscripts utilizing custom algorithms or software that are central to the research but not yet described in published literature, software must be made available to editors and reviewers. We strongly encourage code deposition in a community repository (e.g. GitHub). See the Nature Portfolio guidelines for submitting code & software for further information.

Data

Policy information about availability of data

All manuscripts must include a data availability statement. This statement should provide the following information, where applicable:

- Accession codes, unique identifiers, or web links for publicly available datasets
- A description of any restrictions on data availability
- For clinical datasets or third party data, please ensure that the statement adheres to our policy

Source data are provided with this paper. All other data will be made available from the corresponding author G.C. upon reasonable request. This study did not generate any unique codes.

Research involving human participants, their data, or biological material

Policy information about studies with human participants or human data. See also policy information about sex, gender (identity/presentation), and sexual orientation and race, ethnicity and racism.

Reporting on sex and gender	This study did not use human participants or human data
Reporting on race, ethnicity, or other socially relevant groupings	This study did not use human participants or human data
Population characteristics	This study did not use human participants or human data
Recruitment	This study did not use human participants or human data
Ethics oversight	This study did not use human participants or human data

Note that full information on the approval of the study protocol must also be provided in the manuscript.

Field-specific reporting

Please select the one below that is the best fit for your research. If you are not sure, read the appropriate sections before making your selection.

☒ Life sciences ☐ Behavioural & social sciences ☐ Ecological, evolutionary & environmental sciences

For a reference copy of the document with all sections, see nature.com/documents/nr-reporting-summary-flat.pdf

Life sciences study design

All studies must disclose on these points even when the disclosure is negative.

Sample size	No statistical tests were used to pre-determine sample size, but sample size was chosen based on previously published studies from our group (Stegen S et al, Nature 2019; Stegen S et al, Bone Research 2022). Sample size for each experiment was inferred by the number required for p<0.05 with 80% power, and is indicated in each figure legend.
Data exclusions	No data were excluded.
Replication	All experimental findings were reproduced in multiple independent experiments. For experiments using cultured cells, each independent experiment used osteoclast progenitors isolated from another mouse. For each figure panel, the number of independent experiments or biological replicates is indicated in the figure legends. Data shown in figure panels are the mean of all independent biological repeats. Western blot pictures are from representative experiments and the number of independent repeats is clearly indicated in the figure legends.
Randomization	We did not use statistical methods for randomization.<br>For in vivo experiments, mice were randomly allocated into experimental groups.<br>For in vitro experiments, cells were isolated from randomly chosen wild-type or conditional knockout mice, and randomly allocated to the different treatment groups.
Blinding	Blinding was widely used in the study. Data collection and analysis, such as mouse phenotyping, immunostaining, qRT-PCR, and Western blot were frequently performed by participants other than the experiment designer. During these data collection and analysis steps, all participants were routinely blinded to group allocation.

Reporting for specific materials, systems and methods

We require information from authors about some types of materials, experimental systems and methods used in many studies. Here, indicate whether each material, system or method listed is relevant to your study. If you are not sure if a list item applies to your research, read the appropriate section before selecting a response.

Materials & experimental systems

n/a	Involved in the study
☐	☒ Antibodies
☒	☐ Eukaryotic cell lines
☒	☐ Palaeontology and archaeology
☐	☒ Animals and other organisms
☒	☐ Clinical data
☒	☐ Dual use research of concern
☒	☐ Plants

Methods

n/a	Involved in the study
☒	☐ ChIP-seq
☐	☒ Flow cytometry
☒	☐ MRI-based neuroimaging

Antibodies

Antibodies used

Primary antibodies for Western blot analysis:
C-MYC (Cell Signaling Technologies): #5605 (D84C12, Rabbit); 1/1000 dilution
NFATc1 (Santa Cruz Biotechnologies): sc-7294 (7A6, Mouse); 1/1000 dilution
PHGDH (Cell Signaling Technologies): #66350 (D8F3O, Rabbit); 1/1000 dilution
PSAT1 (Bio-Techne): NBP1-55368 (TL2687305A, Rabbit); 1/1000 dilution
H3K4Me3 (Cell Signaling Technologies): #9751 (C42D8, Rabbit); 1/1000 dilution
H3K9Me3 (Cell Signaling Technologies): #13969 (D4W1U, Rabbit); 1/1000 dilution
H3K27Me3 (Cell Signaling Technologies): #9733 (C36B11, Rabbit); 1/1000 dilution
H3K36Me3 (Cell Signaling Technologies): #4909 (D5A7, Rabbit); 1/1000 dilution
H3K79Me3 (Cell Signaling Technologies): #74073 (E8B3M, Rabbit); 1/1000 dilution
H3 (Cell Signaling Technologies): #14269 (1B1B2, Mouse); 1/1000 dilution
beta-actin (Sigma-Aldrich): A5441 (lot 026M, Mouse); 1/10.000 dilution
Lamin A/C (Santa Cruz Biotechnologies): sc-376248 (lot C1413, Mouse); 1/1000 dilution

Secondary antibodies for Western blot analysis:
anti-mouse HRP conjugated (Dako): P0161 (lot 00095192, Rabbit); 1/2500 dilution
anti-rabbit HRP conjugated (Dako): P0448 (lot 00094764, Goat); 1/2500 dilution

Primary antibody for immunohistochemical analysis:
PHGDH (Cell Signaling Technologies): #66350 (D8F3O, Rabbit); 1/50 dilution

Secondary antibody for immunohistochemical analysis:
anti-rabbit Alexa-fluor 488 (Invitrogen): #A-11034 (lot 1937195, Goat); 1/100 dilution

Antibodies for flow cytometry analysis:
APC-CD11b (#101211; clone M1/70; BioLegend); 1/100 dilution
FITC-Gr-1 (#108405; clone RB6-8C5; BioLegend); 1/100 dilution
FITC-F4/80 (#123107; clone BM8; BioLegend); 1/100 dilution
PE-CD115 (#165203; clone W19330E; BioLegend); 1/100 dilution
PerCP-CD19 (#115531; clone 6D5; BioLegend); 1/100 dilution

Validation

All antibodies were obtained from indicated commercial vendors with ensured quality and have been used in multiple experiments to detect intended proteins in control samples with expected molecular weight to validate their effectiveness in our study. As additional validation, we performed shRNA-mediated knockdown of several genes in vitro.

Antibodies for Western blot/immunohistochemistry:
C-MYC (Cell Signaling Technologies; #5605; D84C12, Rabbit): validated by the manufacturer on HEK293, HeLa, and Raji cells with 888 citations
NFATc1 (Santa Cruz Biotechnologies; sc-7294; 7A6, Mouse): validated by the manufacturer on Raji, Ramos, GA-10, and U-698 with 9 citations
PHGDH (Cell Signaling Technologies; #66350; D8F3O, Rabbit): validated by the manufacturer on HeLa, HT-29, HCT116, and MCF7 cells with 13 citations
PSAT1 (Bio-Techne; NBP1-55368; TL2687305A, Rabbit): validated by the manufacturer on human fetal kidney, and Hep2G cells with 1 citation
H3K4Me3 (Cell Signaling Technologies; #9751; C42D8, Rabbit): validated by the manufacturer on HeLa, and NIH/3T3 cells with 515 citations
H3K9Me3 (Cell Signaling Technologies; #13969; D4W1U, Rabbit): validated by the manufacturer on HeLa, and NIH/3T3 cells with 127 citations
H3K27Me3 (Cell Signaling Technologies; #9733; C36B11, Rabbit): validated by the manufacturer on HCT116, NIH/3T3, C6, COS, and NIH/3T3 cells with 1151 citations
H3K36Me3 (Cell Signaling Technologies; #4909; D5A7, Rabbit): validated by the manufacturer on HeLa, C2C12, C6, and COS cells with 103 citations
H3K79Me3 (Cell Signaling Technologies; #74073; E8B3M, Rabbit): validated by the manufacturer on HeLa, C2C12, C6, and COS-7 cells with 7 citations
H3 (Cell Signaling Technologies; #14269; 1B1B2, Mouse): validated by the manufacturer on HeLa, NIH/3T3, C6, and COS-7 cells with

150 citations
beta-actin (Sigma-Aldrich; A5441; lot 026M, Mouse): validated by the manufacturer on HeLa, JURKAT, NIH/3T3, PC12, RAT2, CHO, MDBK, MDCK, and COS-7 cells with 10021 citations
Lamin A/C (Santa Cruz Biotechnologies; sc-376248; lot C1413, Mouse): validated by the manufacturer on HeLa, C32, NIH/3T3, PC-3, A-431, and Sol8 cells with 229 citations

Antibodies for flow cytometry analysis:
APC-CD11b (#101211; clone M1/70; BioLegend): validated by the manufacturer using C57BL/6 mouse bone marrow cells with 356 citations
FITC-Gr-1 (#108405; clone RB6-8C5; BioLegend): validated by the manufacturer using C57BL/6 mouse bone marrow cells with 127 citations
FITC-F4/80 (#123107; clone BM8; BioLegend): validated by the manufacturer using Balb/c mouse peritoneal macrophages with 207 citations
PE-CD115 (#165203; clone W19330E; BioLegend): validated by the manufacturer using Balb/c mouse peritoneal macrophages with 0 citations
PerCP-CD19 (#115531; clone 6D5; BioLegend): validated by the manufacturer using mouse splenocytes with 17 citations

Animals and other research organisms

Policy information about studies involving animals; ARRIVE guidelines recommended for reporting animal research, and Sex and Gender in Research

Laboratory animals	All mice that were used in this study were on a C57BL/6J genetic background. Osteoclast-specific PHGDH-deficient mice were generated by crossing Phgdh-floxed mice (Yoshida K, J Biol Chem 2004) with transgenic mice expressing the Cre recombinase under the control of the Lysozyme M gene promotor (Clausen BE, Transgenic Res 1999). Wild-type littermates (Phgdh-floxed mice without expression of the Cre recombinase) were used as control. All mice were individually genotyped and correct excision of floxed alleles was determined. Mouse phenotyping was performed on 8-week-old mice. All colonies were housed and bred in individually ventilated cages in the animal facility of the KU Leuven (at 18-23°C and 40-60% humidity with a 12 hours light-dark cycle).
Wild animals	This study did not use wild animals.
Reporting on sex	The in vivo impact of Phgdh deletion in osteoclasts was confirmed both in male and female mice. Specifically, mouse phenotyping was performed in 9 wild-type and 7 conditional knockout male mice (microCT and histological analysis), and 6 wild-type and 9 conditional knockout female mice (microCT analysis). For pharmacological PHGDH inhibition, we used either male mice (vehicle/NCT-503; n=5) or sham-operated/ovariectomized female mice (vehicle/NCT-503; n=6). All in vivo experiments, regardless of sex, consistently indicate that inactivation of PHGDH results in decreased osteoclast-mediated bone resorption and consequently increased bone mass.
Field-collected samples	This study did not include field-collected samples.
Ethics oversight	All experimental procedures were approved by the Institutional Animal Care and Research Advisory Committee of the KU Leuven (protocol number P140/2020).

Note that full information on the approval of the study protocol must also be provided in the manuscript.

Flow Cytometry

Plots

Confirm that:

☒ The axis labels state the marker and fluorochrome used (e.g. CD4-FITC).

☒ The axis scales are clearly visible. Include numbers along axes only for bottom left plot of group (a 'group' is an analysis of identical markers).

☒ All plots are contour plots with outliers or pseudocolor plots.

☒ A numerical value for number of cells or percentage (with statistics) is provided.

Methodology

Sample preparation	Bone marrow myeloid cells were obtained by flushing tibiae and femurs with glutaMAX-1 aMEM, supplemented with 100 units/ml penicillin, 50 µg/ml streptomycin and 10% fetal bovine serum, and enriched for CD45 expression using CD45 MicroBeads (Miltenyi Biotec) and magnetic-activated cell sorting according to the manufacturer's instructions. Cells were spun down and labeled with the following antibodies for 45 minutes at 4°C: APC-CD11b (#101211, BioLegend), FITC-Gr-1 (#108405, BioLegend), FITC-F4/80 (#123107, BioLegend), PE-CD115 (#565249, BioLegend) and PerCP-CD19 (#115531, BioLegend).
Instrument	BD Canto II HTS
Software	Data acquisition: BD FACSCanto II system software v3.0<br>Data analysis: Kaluza 2.2.1 software

Cell population abundance	Post-sort purity was not determined
Gating strategy	Gating strategy is provided as Extended Data Fig. 3m. Gates were determined based on single-label beads (AbC Total Antibody Compensation Bead Kit; FisherScientific) and negative controls (non-labeled beads).

☒ Tick this box to confirm that a figure exemplifying the gating strategy is provided in the Supplementary Information.
