# Supplementary figures and images for: The serine synthesis pathway drives osteoclast differentiation through epigenetic regulation of NFATc1 expression
Source: Nat Metab. 2024 Jan 10;6(1):141–52. doi: 10.1038/s42255-023-00948-y (PMC10822776; doi:10.1038/s42255-023-00948-y)

Fig. 1k-l

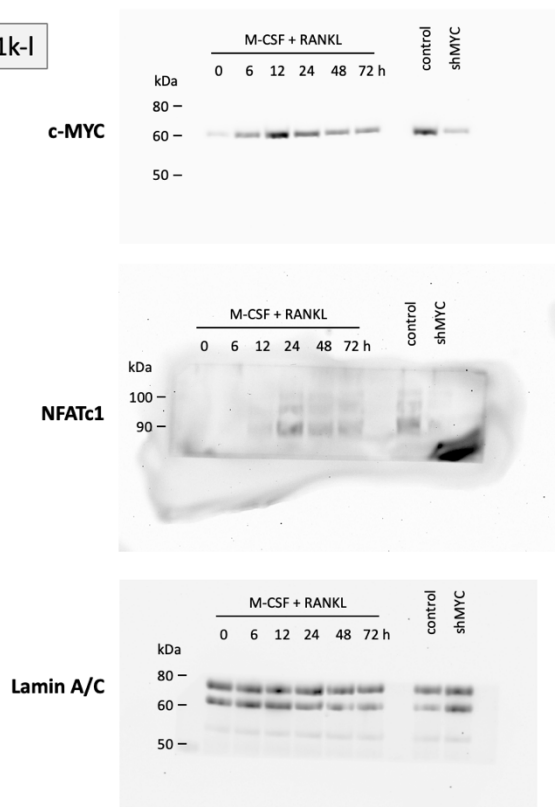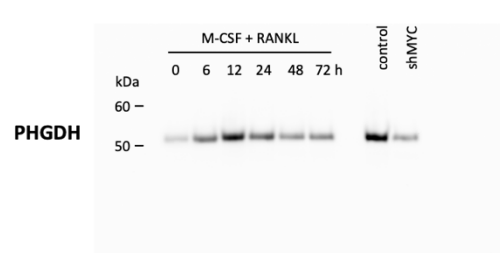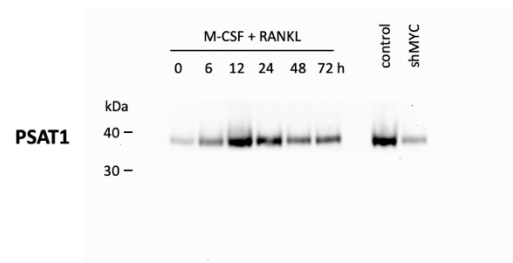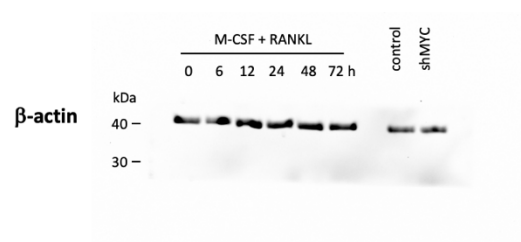

Fig. 5f

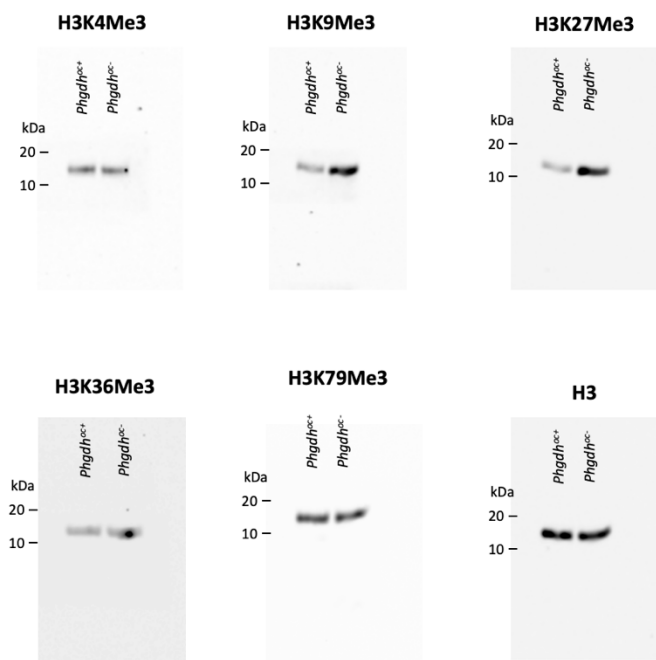

Fig. 5h

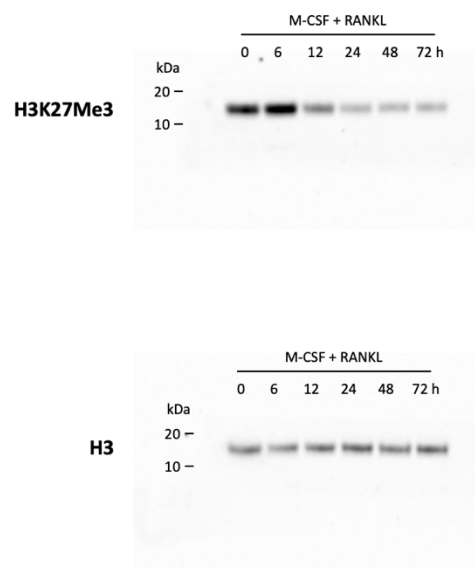

Fig. 5i

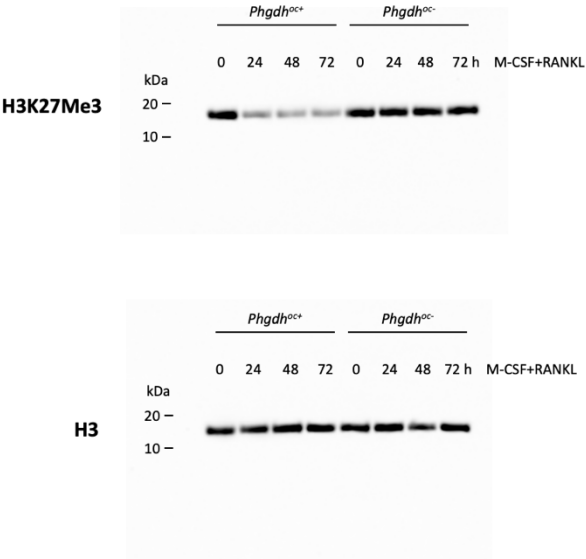

Fig. 5j

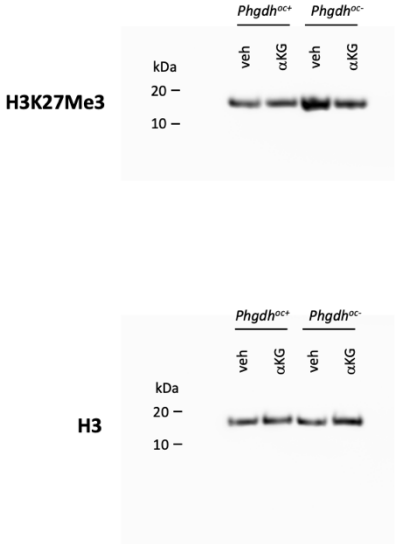

Extended Data Fig. 3b

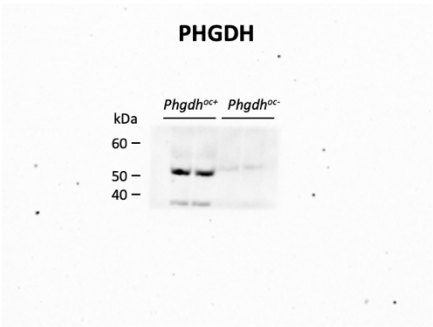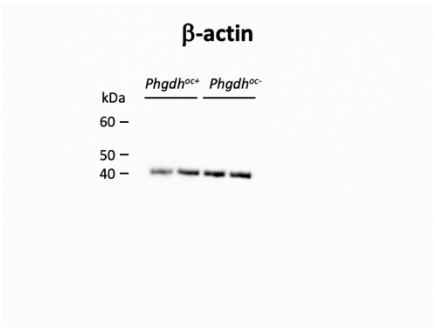

Supplement: Supplementary file 20 — Uncropped western blots of both main figures and Extended Data figures. [file 42255_2023_948_MOESM20_ESM.pdf]
